# Unraveling the clonal hierarchy of somatic genomic aberrations

**DOI:** 10.1186/s13059-014-0439-6

**Published:** 2014-08-26

**Authors:** Davide Prandi, Sylvan C Baca, Alessandro Romanel, Christopher E Barbieri, Juan-Miguel Mosquera, Jacqueline Fontugne, Himisha Beltran, Andrea Sboner, Levi A Garraway, Mark A Rubin, Francesca Demichelis

**Affiliations:** 1 Centre for Integrative Biology, University of Trento, Povo Trento, 38123 Italy; 2 The Broad Institute of MIT and Harvard, Cambridge, 02141 MA USA; 3 Dana-Farber Cancer Institute, Boston, 02215 MA USA; 4 Harvard Medical School, Boston, 02115 MA USA; 5 Department of Urology, Weill Medical College of Cornell University, New York, 10065 NY USA; 6 Department of Pathology and Laboratory Medicine, Weill Medical College of Cornell University, New York, 10065 NY USA; 7 Department of Medicine, Division of Hematology and Oncology, Weill Medical College of Cornell University, New York, 10065 NY USA; 8 Institute for Precision Medicine, Weill Medical College of Cornell University and New York Presbyterian Hospital, New York, 10065 NY USA; 9 HRH Prince Alwaleed Bin Talal Bin Abdulaziz Alsaud Institute for Computational Biomedicine, Weill Medical College of Cornell University, New York, 10065 NY USA

## Abstract

Defining the chronology of molecular alterations may identify milestones in carcinogenesis. To unravel the temporal evolution of aberrations from clinical tumors, we developed CLONET, which upon estimation of tumor admixture and ploidy infers the clonal hierarchy of genomic aberrations. Comparative analysis across 100 sequenced genomes from prostate, melanoma, and lung cancers established diverse evolutionary hierarchies, demonstrating the early disruption of tumor-specific pathways. The analyses highlight the diversity of clonal evolution within and across tumor types that might be informative for risk stratification and patient selection for targeted therapies. CLONET addresses heterogeneous clinical samples seen in the setting of precision medicine.

**Electronic supplementary material:**

The online version of this article (doi:10.1186/s13059-014-0439-6) contains supplementary material, which is available to authorized users.

## Background

Cancer arises from initiating cells (clones) that undergo intense evolutionary selection during disease progression and can be widely altered during treatment. The tumor cell evolutionary process may lead to subclonal divergence resulting in genetic and molecular heterogeneity. Computational approaches to establish maps of cancer evolution might inform clinical risk stratification and treatment strategies. Analysis strategies have been developed to address tumor DNA purity and cancer cell ploidy, but there remains a gap for the analysis of minimally aberrant or highly heterogeneous tumors.

Over the past years, several methods have been developed to quantify DNA admixture and ploidy from high density single-nucleotide polymorphism (SNP) array data [1]-[4] that utilize the relative abundance of specific allele signal (B allele frequency (BAF)) and the tumor over normal signal ratio (referred to as Log R) to measure the complexity of the underlying cellular population. Global optimization methods are applied to find the configuration of DNA admixture and ploidy that better account for the observed values of BAF and Log R. More recent tools [5]-[9] exploit the rich statistical properties of massively parallel sequencing to provide base-pair data resolution. Using germline heterozygous SNP loci (hereafter called informative SNPs), tumor purity and ploidy are estimated analyzing allelic fraction (AF) values (that is, the fraction of sequencing reads supporting the reference base) in a way resembling the use of BAF data in SNP arrays. Subclonal alterations will appear as outliers from the computed admixture and ploidy.

All these tools apply a global approach (that is, the AF (or BAF) values are all thrown into an inference algorithm that eventually returns DNA purity and ploidy). Global methods are well-suited for tumor samples with fairly homogenous genomic aberrations (high ratio of clonal versus subclonal lesions). In the clinical setting, where tumor samples might exhibit heterogeneity due to progression or subsequent to multiple lines of treatment, and for tumor types that undergo structural changes such as `chromoplexy’ events in prostate cancer (that is, abundant DNA rearrangements and deletions that arise in a highly interdependent manner) [10], these approaches may prove suboptimal as they ignore the genomic diversity.

**Figure 1:**
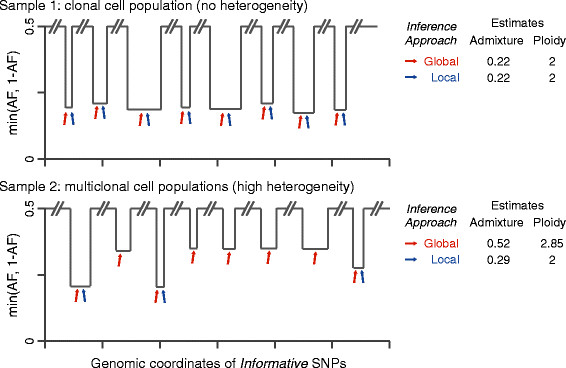
**Local versus global approaches to clonality analysis.** The local optimization approach to compute global admixture (1 - purity) and ploidy is schematically explained and compared with a global optimization approach on two cancer samples with different levels of lesion heterogeneity. Copy number neutral regions show nominal AF of 0.5; AFs of mono-allelic deletions depend on lesion clonality and on global admixture. Arrows point to lesions used by global (red) or local (blue) approach. For sample 1 showing a profile compatible with clonal cell population (that is, each lesion demonstrates approximately the same AF value), the two approaches return equivalent estimates. For sample 2 (high heterogeneity) the local approach focuses on lesions with the highest level of clonality (blue arrows), resulting in a realistic estimate of the sample admixture.

These observations prompted us to develop a second generation tool based on local (in contrast to global) optimization where estimates of purity and ploidy are derived from few clonal events (Figure 1). We noted that the AF values of the informative SNPs within a somatic deletion result from the composition of signal from three cell populations: (i) non-tumor cells (contributing to the DNA admixture); (ii) tumor cells without the deletion; and (iii) tumor cells harboring the deletion (that is, a subclonal deletion given (ii) and (iii)). By modeling the probability distribution of the observed AFs, we compute a local estimate of the DNA admixture (1 - DNA purity) that accounts for both normal cell admixture and subclonal tumor cell population. After estimating local admixture values for all deletions across the genome, only selected lesions (from the most clonal side of the spectrum) contribute to the computation of the tumor sample global admixture. In the presence of homogenously aberrant genomes (Figure 1, top), global and local approaches result in similar estimates; for heterogeneous genomes (Figure 1, bottom), the local approach focused on selected lesions (blue arrows in Figure 1) leads to more realistic estimates. Here, we present the full implementation of CLONET (CLONality Estimate in Tumors) and study the clonality of somatic aberrations from whole genome sequencing (WGS) data across 3 tumor types comprising 55 individuals with primary prostate cancer [10], 24 metastatic melanomas [11], and 21 lung adenocarcinomas [12].

## Results

### Clonality assessment of aberrations from sequencing reads

We reasoned that the reads mapped into a genomic window can be partitioned in two sets: one set includes reads that equally represent parental chromosomes (copy number neutral reads); and the other set contains reads from only one parent chromosome (active reads). There are four main steps that, starting from neutral read counts, allow inference of clonality of any genomic window. First, we estimate the percentage of neutral reads within a genomic segment independently of its Log R value. Second, we use the Log R value to relate the neutral reads percentage with a local estimate of DNA admixture. Local estimates are then aggregated to estimate global admixture and clonality of somatic copy number aberrations (SCNAs). Third, aneuploidy genomes are identified and the analysis corrected accordingly. Finally, we extend the analysis to point mutations (PMs) and structural rearrangements (REARRs) in a coherent manner. In the following we will briefly detail each step.

**Figure 2:**
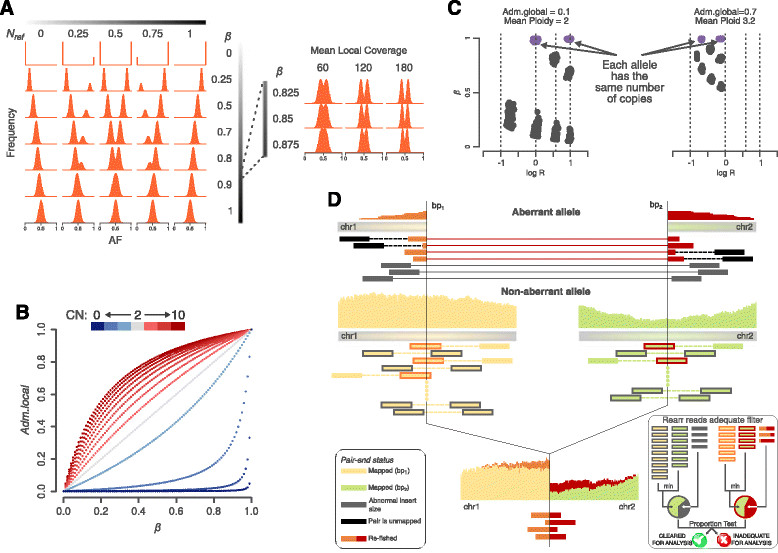
**CLONET key elements. (A)** For each genomic segment, the AF distribution of informative SNPs follows a bimodal distribution that depends on the proportion of reference bases in the active allele (N_ref_) and on the percentage of neutral reads (*β*) (left panel; 60X coverage data used For high values of *β* the distribution becomes unimodal as a function of the coverage (right panel; N_ref_ = 0.5). **(B)** For a genomic segment, the relationship between *Adm.local* and *β* is a hyperbola whose parameter values are governed by the segment copy number (CN). **(C)** Genomic segment representation in the *β* versus Log R space depends on the combination of the copy number of the parental alleles. Aneuploidy causes a shift while *Adm.global* results in shrinkage of the allowed subspaces. Violet dots indicate genomic segments with equal number of parental alleles (that is, 1/1, 2/2, 3/3,…). **(D)** The sketch illustrates the alternative allele proportion (AP) of a mono-allelic REARR defined by the genomic coordinates bp_1_ and bp_2_ The aberrant allele is represented by two types of reads: reads with abnormal insert size (gray) and reads that span both sides of a breakpoint (orange for bp_1_ and red for bp_2_). The non-aberrant allele exhibits two matching classes: paired-end crossing reads where one end maps entirely to one side and the other entirely to the other side correspond to aberrant gray reads. Single end reads that span the breakpoint (overlapping) are a complementary match to aberrant orange and red reads. The clonality is proportional to the proportion of aberrant reads around the breakpoint junction. *Ad hoc* quality filters are applied for removing sequencing artifacts around REARRs (right inset). First, the non-aberrant allele coverage is the minimum between the coverage in bp_1_ and bp_2_. Then, a REARR is adequate for analysis if the AP is statistically indistinguishable if we consider overlapping or crossing reads (proportion test with limit value 0.1).

For each genomic segment *Seg*, the expected AF of the informative SNP in *Seg* has bimodal distribution that relates to the composition of the DNA sample; the distance between the two modes is proportional to the percentage of neutral reads *β* (see Materials and methods). The expected distribution of the AF (Figure 2A) varies accordingly with *β* and *N*_ref_, which is the proportion of reference base reads in the allele represented by active reads. For each input segment *Seg*, optimization based on swarm intelligence [13] finds a *β* that minimizes the difference between the expected and the observed AF distribution (see Materials and methods). Then, the Log R of *Seg* allows computing a local estimate of the admixture. If *Seg* defines a mono-allelic deletion, *β* corresponds to the percentage of reads deriving from cells that do not harbor the deletion (see Materials and methods) and relates to a local estimate of the percentage of admixed cells, *Adm.local*, as (proof in Additional file 1):

1 $$\mathrm{Adm}.\mathrm{local}=\;\frac{\beta }{2\beta \beta }$$

Reasoning that the signal from normal cells is uniform along the genome, local admixture values are then clustered, and the lowest median value among all the clusters determines the global admixture (*Adm.global*) of the sample. Reasoning that the more the local admixture value differs from the global one the more *Seg* is subclonal, the clonality of *Seg*, *Cl*_*Seg*_, is computed as the percentage of tumor cells in a sample harboring *Seg.* If *Seg* is a gain, Equation 1 extends by rescaling the percentage of neutral reads *β* to recover the percentage of reads sequenced from cells that does not harbor the gain of *Seg* (Figure 2B; Materials and methods). Bi-allelic deletions are treated separately; if the deletion is clonal, its AF distribution has binomial distribution (*β* = 1) and represents only DNA admixture, but in case of subclonality, the value of *β* is proportional to the percentage of tumor cells that do not harbor the deletion (Additional file 2).

Aneuploidy causes a shift in the Log R versus *β* space of a sample (Figure 2C). In any genomic segment with an empty active reads set (neutral copy number segment) each allele has the same number of copies, and its *β* is 1 by definition. The ploidy of a sample is the shift in the Log R values of the neutral segment that best accounts for the observed Log R values. Log R data are then corrected for both ploidy and *Adm.global* to achieve better estimates of the segment copy number (Figure S2A-C in Additional file 3).

Clonality estimates of PMs build on the assumption that reads supporting the alternative allele are representative of the amount of tumor DNA harboring the mutation. In particular, the proportion of reads supporting the alternative allele (AP) of a 100% pure and clonal hemizygous PM has symmetric binomial distribution. The *Adm.global* value represents the percentage of reads from admixed cells that have to be ignored to compute the correct value of the AP. A PM is subclonal when its corrected AP has a low probability to be clonal (type I error < 0.05). The same principle applies to REARRs by properly selecting reads from rearranged tumor cells. The total number of reads that span both sides of a breakpoint defining a REARR [14] is a proxy of the number of cells harboring the rearrangement (Figure 2D). After removing reads representative of global admixture, the difference between the expected and the observed proportion of reads supporting the alternative allele is proportional to the subclonality of the considered REARR (Additional file 1).

### Inferring the order of mutations in a tumor sample

**Figure 3:**
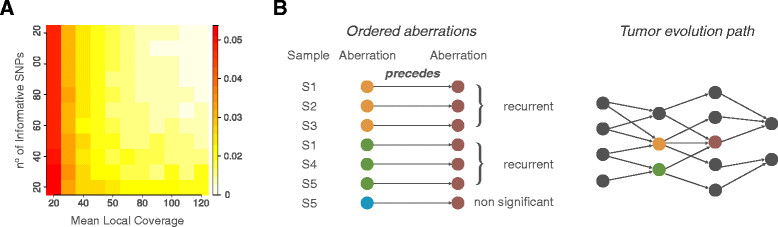
**Building the evolution path. (A)** For each genomic segment, the uncertainty value around *β* is a function of the number of informative SNPs and of the mean local coverage. **(B)** Tumor evolution paths are built from ordered aberrations consistently observed in multiple samples. On the left, arrows indicate ordered aberrations based on clonality estimates within the same tumor; three distinct aberrations (orange, green, and blue) precede a fourth one (red). Ordered aberrations that find statistical support (as recurrent in multiple samples) contribute to the tumor evolution path (right).

The assessment of the clonality of each somatic aberration enables the deconvolution of the sequence of oncogenic events that occur during tumor initiation or progression. Assuming that clonal alterations originated prior to subclonal alterations within the same tumor, we examined pairs of genes that are aberrant in the same sample and across multiple tumors to determine the directionality of the clonal-subclonal hierarchy. However, different error sources may introduce a bias into the distribution of the AF that could lead to inaccurate clonality estimates. To minimize the number of false positives (clonal aberrations called subclonal), we compute the estimation uncertainty around β (Figure 3A) and propagate it to clonality values (see Materials and methods). Error management enables robust comparison of aberration clonality across different tumor sample data. We then apply the following algorithm to determine the progression on somatic aberrations: if a clonal aberration A_1_ and a subclonal aberration A_2_ co-occur within the same sample S, we assume that A_1_ has been acquired before A_2_ in S and we say that A_1_ precedes A_2_ in S. To then derive the rule that links aberration A_1_ and A_2_, the same dependency has to be found consistently across many samples. This strategy can produce an evolution path draft (that is, a pictorial representation of the potential temporal relations among somatic aberrations observed in a sample set (Figure 3B)). In the presence of adequate sample size and frequencies of co-occurring aberrations, the statistical significance of the relation between A_1_ and A_2_ can be assessed by testing the null hypothesis that two aberrations are independent (that is, A_1_ precedes A_2_ or A_2_ precedes A_1_ are equally likely) and consider a binomial distribution *B*(*n*,*p*) with number of trials *n* equals the number of samples where A_1_ is clonal and A_2_ is subclonal or vice versa, and success probability *P* = 0.5 (binomial test with 5% significance would require a minimum of 6 out of 6 samples where A_1_ precedes A_2_).

### *In silico* and *in situ*experimental validation

To assess if the coverage depth typical of large scale sequencing experiments (around or below 100X for WGS) has an effect on clonality estimates of SCNAs or PMs, we first queried MiSeq ultra-deep sequencing (approximately 65,000X) data generated from a set of 18 aberrant genes from 7 tumor samples analyzed in this study and observed excellent agreement in downstream clonality calls for deletions (Cochran test, *P* = 1) [10]. Notably, CLONET did not assign clonality values to aberrations in which MiSeq does not confirm WGS-based AP values. Next, we focused on a set of study PMs and assessed high correlation of AP values between WGS and MiSeq (mean coverage >143,000X) data (Figure S3A in Additional file 4; Table S1 in Additional file 5) (Pearson’s r = 0.73, *P* < 10e-3), suggesting altogether that the study coverage does not significantly impact the ability to assess aberration clonality.

**Figure 4:**
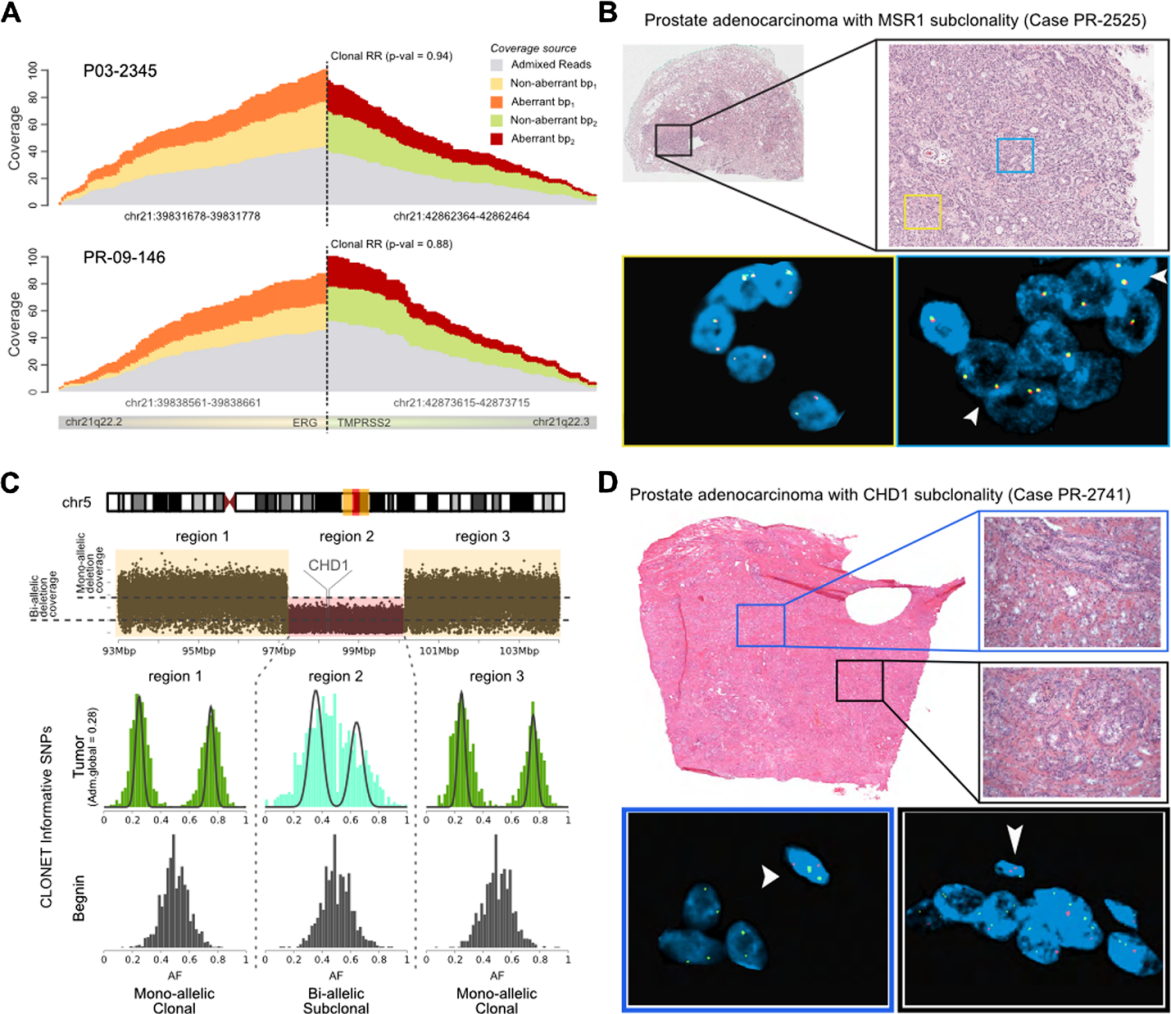
***In silico*** **and** ***in situ*** **validation. (A)** Coverage of overlapping aberrant and non-aberrant reads at the REARR breakpoints of clonal *TMPRSS2-ERG* REARRs in two prostate adenocarcinomas (cases P03-2345 and PR-09-146). Upon removal of admixed DNA reads (gray), local coverage of aberrant and non-aberrant reads match (AP close to 0.5), supporting the clonality of the rearrangement. **(B)** Representative case (PR-2525) with *MSR1* subclonality REARR, validation by FISH*.* Low power view of prostate adenocarcinoma, Gleason score 3 + 4 = 7 in a prostatectomy specimen (black box). Some areas do not have deletion of *MSR1* as demonstrated by the presence of two yellow signals in tumor nuclei (yellow box). Other areas show hemizygous deletion of *MSR1* as demonstrated by the presence of only one yellow signal in tumor nuclei (blue box). Occasional nuclei with wild-type *MSR1* (arrow heads) are identified in this area. Nuclei with *MSR1* hemizygous deletion comprised approximately 30% of assessed tumor areas. **(C)** A homozygous subclonal deletion including *CHD1* is inserted within a large hemizygous clonal deletion (case PR-2741)*.* Cancer AF highlights the different proportion of aberrant reads in the two cases. **(D)** Representative case with *CHD1* subclonality (case PR-2741), validation by FISH. Low power view of prostate adenocarcinoma, Gleason score 4 + 3 = 7 in a prostatectomy specimen. Some areas have homozygous deletion of *CHD1* as demonstrated by the presence of only two green signals (reference probe) in tumor nuclei (bottom left blue box). In contrast, other areas show hemizygous deletion of *CHD1* as demonstrated by the presence of one red (*CHD1*) and two green signals (reference probe) in tumor nuclei (bottom right black box). Note the presence of two red and two green signals (wild-type *CHD1*) in adjacent stromal cells, used as internal control (arrow heads).

In order to validate the clonality status of more complex structural genomic aberrations, specifically gene rearrangements and homozygous deletions, we turned to *in situ* tests on human tumor samples. As proof of principle, we first demonstrated the ability to assess rearrangement clonality focusing on well-characterized prostate-specific clonal REARRs involving *ERG* and *TMPRSS2*[15]. Separate clonality analyses of both *TMPRSS2*-*ERG* REARRs and of the accompanying 3 Mb interstitial deletion [16] in two prostate tumors (P03-2345 and PR-09-146) demonstrated perfect agreement (Figure 4A; Figure S3B in Additional file 4). Next, we validated by fluoresce *in situ* hybridization (FISH) analyses two subclonal REARR calls, involving a genomic area 86 kb upstream of *MSR1* (8p22) in patient PR-2525 (Figure 4B) and *SPRY2* (13q31.1) in patient PR-3042 (Figure S4A in Additional file 6).

Finally, we validated *in situ* a homozygous deletion along 5q spanning *CHD1* that was predicted to be subclonal. Figure 4C shows the sequencing data information utilized by CLONET to infer the subclonal bi-allelic deletion in patient sample PR-2741 as confirmed by FISH (Figure 4D). The prediction highlights a relatively small subclonal bi-allelic deletion (Figure 4C, region 2) within a larger clonal mono-allelic deletion (Figure 4C, regions 1 and 3), suggesting that selective evolutionary pressure is acting on the genomic region. Patient PR-2525 demonstrated a similar subclonality pattern at the *CHD1* locus (Figure S4B in Additional file 6).

### Comparison of CLONET and ABSOLUTE

We compared *Adm.global* predictions for prostate cancer and melanoma samples with calls reported in the original manuscripts [10],[11] (data for lung not publicly available) using a widely used computational method, ABSOLUTE [5], that implements a global estimation approach. Overall agreement was observed (Figure S5A, B in Additional file 7) in both datasets (Pearson’s r ≥ 0.8, *P* < 10e-5). Not surprisingly, CLONET allowed for larger fractions of admixture calls compared with ABSOLUTE (98% versus 74% in prostate and 92% versus 88% in melanoma) as the local approach better handles samples with low ratios of clonal to subclonal SCNAs. The average ratio across cases only handled by CLONET is 1.73 versus average ratios across cases handled by both of 5.72 and 6.88 for prostate and melanoma samples, respectively. We further tested CLONET performance on whole exome sequencing (WES) data using an independent cohort of 108 prostate samples [17] (Pearson’s r = 0.74, *P* < 10e-15; Figure S5C in Additional file 7).

In terms of ploidy assessment we noted significant differences in the melanoma dataset where CLONET tends to undercall polyploidy (Figure S5D in Additional file 7). Where our conservative approach might definitely introduce false negative calls, we identified cases where close inspection of allele-specific data is not necessarily compatible with ABSOLUTE original calls of polyploidy [11]; for example, ME049T (Figure S5E in Additional file 7), where the relative distances among Log R peaks are more compatible with a diploid genome.

### Comparative analysis reveals different mechanisms of tumor deregulation

**Figure 5:**
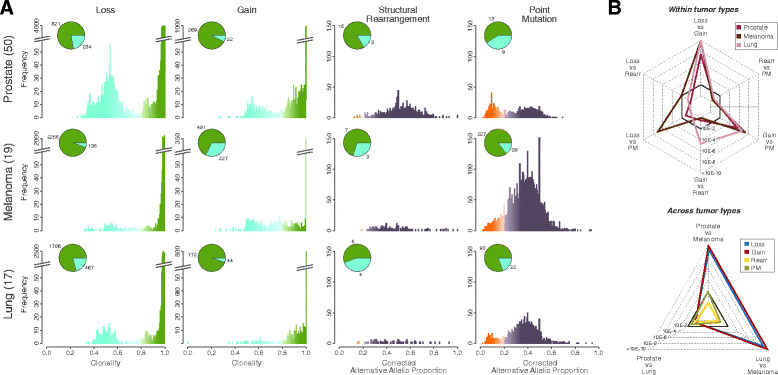
**Clonality comparison across somatic aberrations and tumor types. (A)** Summary of clonality: clonality inference of genomic events. Pie charts indicate the mean numbers of events classified as clonal (green) or subclonal (blue) across samples. **(B)** Statistics of clonality: within tumor type and across tumor type comparisons of the proportion of the mean number of clonal/subclonal genomic events. Radar charts report the statistics of the comparisons (*P*-values of the proportion test with Benjamini-Hochberg FDR correction). Dark gray continuous line represents 99% confidence level.

We analyzed a total of 17,645 losses, 4,753 gains, 7,728 PMs, and 1,504 REARRs (Figure S6A in Additional file 8) and a panel of more than 23,000 genes from the RefSeq database [18]. Figure 5A summarizes the distribution of clonality across tumor types and aberrations. We compared the mean number of events classified as clonal or subclonal by means of the proportion test with Benjamini-Hochberg false discovery rate (FDR) correction (Figure 5B; Table S2 in Additional file 5). We observed that deletions are more heterogeneous than gains in prostate and lung cancer (corrected *P*-values <10e-6 and <10e-21, respectively) and, interestingly, melanoma samples showed the opposite behavior (corrected *P*-value <10e-21). Moreover, when comparing the proportion of clonal/subclonal losses and gains across tumor types, the prostate and lung samples are statistically indistinguishable (corrected *P*-values of 0.49 and 0.365, respectively). Overall, this suggests that temporally distinct mechanisms lead to loss and gain across the three tumor types. In terms of PMs, prostate cancer exhibits more subclonal events than melanoma, suggesting a more central role of PMs in melanoma oncogenesis compared with prostate cancer, as confirmed on an independent cohort from The Cancer Genome Atlas (TCGA) of 264 melanoma samples (Figure S6B in Additional file 8). However, aggregated values reflect only part of the story as great variability in the percentage of clonal events within a single combination of tumor and aberration is observed (Figure S6C in Additional file 8). We found no association between the patients’ clinical characteristics and exhibited clonal and subclonal range (Table S3 in Additional file 5).

**Figure 6:**
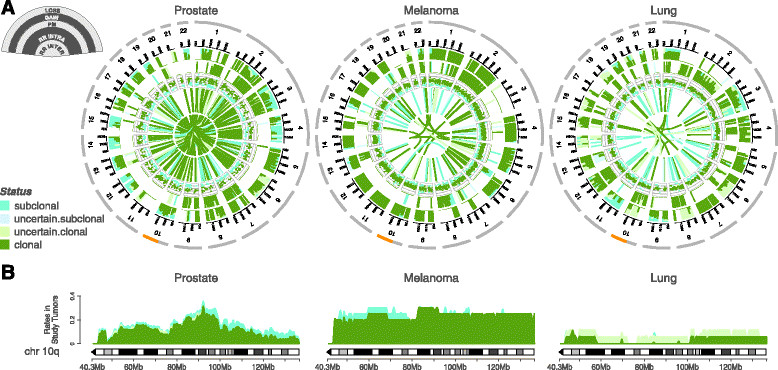
**Clonality distribution along tumor genomes. (A)** For each tumor type the circos plot represents the distribution of clonality along the genome. Each circos plot has five data tracks. The two outermost tracks report the proportion of clonal/subclonal losses and gains, respectively. Then, PM APs and the associated clonality status are depicted in the middle track. Finally, the inner tracks show the clonality status of intra- and inter-chromosomal REARRs. **(B)** Comparison of the clonality status of losses along chromosome 10q across tumor types. For each tumor type, the clonality status of losses was sampled every 100 kb and the proportion of clonal/subclonal losses reported.

Having assessed the variability in the clonality status of aberrations in individual patients, we then evaluated how it distributes along the whole genome as represented in clonality circos plots (Figure 6A). We can appreciate commonality among the three tumor types in some specific genomic regions. Genes on 8p are found clonally deleted in 96%, 100%, and 100% of the prostate, melanoma, and lung samples, respectively. They include the prostate cancer suppressor *NXK3-1*[19], the gene *CSMD1*, which is recurrently deleted in melanoma [11], and the phosphatase *DUSP4*, which is involved in negative feedback control of *EGFR* signaling in lung adenocarcinoma [20].

Then, we investigated whether clonality analysis can highlight tumor-specific mechanisms of deregulation. Our comparative analysis shows that although *PTEN* deletion is involved in many cancer types, the underlying timing of alteration may be different among tumor types (Figure 6B), and may point to differential roles for pathway inactivation. Specifically, the focal and subclonal deletion in the prostate samples suggests that evolutionary pressure is acting in this region later in the natural history of the disease and may promote cancer progression at a later stage of cancer evolution. In contrast, the broad and clonal deletion of a large part of chromosome 10q found in melanoma indicates that *PTEN* is homogenously lost in metastatic melanoma. In lung adenocarcinomas loss of *PTEN* expression in *EGFR*-mutant cells correlates with increased drug resistance [21], but the rarity of the deletion observed entails the hypothesis that loss of expression is not due to a genomic alteration of *PTEN*.

Finally, our tool can also identify tumor lineage-specific subclonality. *MUC4*, encoding a protein acting to upregulate cell cycle inhibitor p27 through ERBB2 phosphorylation [22] and mutated in melanoma [11], is subclonal in the majority of samples (7 out of 12, 58%) on a background mean subclonality rate of 14% (Figure 5A) (adjusted binomial test *P*-value <0.05); this is similar for *MUC2* and *PCMTD1*. The *SOX2* gene, which encodes a transcription factor relevant to lung development and has been identified as a lineage-survival oncogene in lung squamous cells carcinomas [23] and over-expressed in advanced tumors [24], is detected as a subclonal gain in one (case LUAD-AEIUF) of two amplified samples, with possible implications for prognosis and risk stratification.

### Clonal hierarchy of genomic aberrations

**Figure 7:**
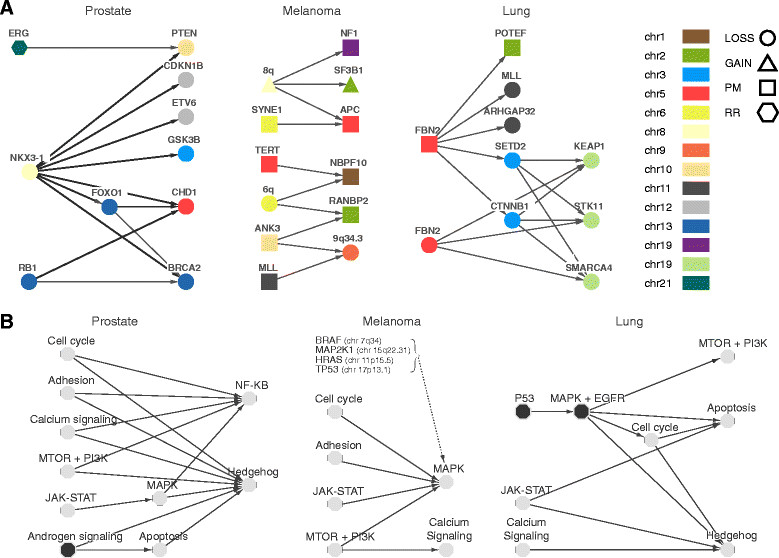
**Observed tumor patterns. (A)** Draft tumor evolution paths of putative driver aberrations based on recurrent patterns. Thicker arrows in the prostate path correspond to precedence relations that were confirmed in an independent cohort of 203 WES prostate samples. **(B)** Draft tumor evolution paths based on cancer pathways. Each node represents a set of genes (aka a pathway). For a pair of pathways P_1_ and P_2_, CLONET computes the number of dependencies from each gene in P_1_ to each gene in P_2_, d_12_, and the inverse, d_21_. If there is no dependency between P_1_ and P_2_, then it is expected that d_12_ and d_21_ are statistically comparable (that is, d_12_ is generated from a symmetric binomial distribution with d_12_ + d_21_ trials). If d_12_ > d_21_ and the *P*-values of the binomial test with Benjamini-Hochberg FDR correction is <0.01, then the arc is reported.

We next analyzed the temporal evolution of driver aberrations [25],[26] to build evolution maps (Figure 7A) capitalizing on the information from multiple individuals’ samples in the absence of multiregion samples from the same individual [27]. Given the study sample size and the mutation frequencies, we built drafts of evolution maps by implementing the following rules. An arrow from aberration A_1_ to aberration A_2_ is drawn if (i) A_1_ and A_2_ co-occur in at least two samples, (ii) A_1_ preceded A_2_ in at least one sample, and (iii) A_2_ does not precede A_1_ in the considered dataset (Figure 7A).

Extended analysis of the prostate tumors for which deletion evolution paths were provisionally characterized [10] confirmed the temporal relation between the deletion of *PTEN* and *TMPRSS2*-*ERG* rearrangements (both via deletions and insertions in patient P01-28) in a coherent analysis framework. In addition, the improved sensitivity of CLONET now identifies additional genes whose loss precedes the homozygous deletion of *CHD1*, namely *NKX3-1*, *FOXO1*, and *RB1* (Figure 7A). To find support for the observed relations, we further analyzed 203 independent localized prostate cancer samples that underwent WES, including the Barbieri *et al*. dataset [17] and the interim TCGA prostate cancer cohort. We verified the vast majority of the relations depicted in Figure 7A (exceptions are *BRCA2* with *FOXO1* and *RB1*). It is worth mentioning that no contradictory relations were detected. In addition, in a set of patients treated with brachytherapy [28], we verified through FISH analysis that *PTEN* subclonal deletion is preferentially observed in the setting of *ERG* rearrangement in line with other recent studies [29].

By querying recently reported gene sets [30],[31] and high confidence driver genes [32], the melanoma sample analysis revealed that clonal gains over chromosome 8q, including the proto-oncogene *MYC*, precede a missense mutation of *NF1*, a negative regulator of *RAS* signaling [30], and that deletions on 6q spanning the pro-apoptotic factor *BCLAF1* (Bcl2-associated factor 1) consistently precede missense mutations of *NBPF10* (chromosome 1q21.1) and *RANBP2* (chromosome 2q12.3).

Similarly, we considered 32 high confidence driver genes [32] in the lung adenocarcinoma set (Figure 7A). CLONET identified a path that stems from the clonal aberration of gene *FBN2*[33], either via deletion or missense mutation. *FBN2* disruption precedes the subclonal deletion of genes along chromosome 9p, including significantly mutated known lung adenocarcinoma genes *SMARCA4*, *KEAP1*, and *STK11*[12]. Interestingly, two paths involving different aberration mechanisms appear to lead to 9p deletion: (i) a direct path when the *FBN2* gene is deleted; and (ii) an indirect one through the loss of the 3p21.31-22.1 genomic region in the case of a missense mutation of *FBN2*.

In order to investigate common patterns of progression across tumor types, we interrogated a large set of putative cancer genes (N = 507; September 2013 version of COSMIC cancer gene census [34]) and applied pairwise intersections (three supporting samples across the two tumor types) of identified paths (Additional file 9). Despite the conservative approach and the overall limited number of samples, we observed that *RB1* loss is consistently sequential to 6q losses in both melanomas and prostate cancers. Similarly, aberrations along 17q (*TP53*) are followed by *NFKB2* loss, supporting additional oncogenic effects [35]. The same cluster of 17q gene aberrations precedes deletions along 12q24 in both prostate and lung samples. Last, we detected consistent subclonal deletions on 9q in both melanoma and lung samples following deletions on 15q and *IRF4* gain. Notably, the path included a subclonal deletion of the tumor suppressor *TSC1* (tuberous sclerosis 1) that may activate the mammalian target of rapamycin (mTOR) pathway and promote tumor development [36].

Finally, we explored the evolution of known cancer signaling pathways (Figure 7B); both common themes across tumor types and tissue-specific patterns emerged. Well-established, recurrently deregulated pathways were detected as early drivers, such as androgen signaling in prostate cancer, core mitogen-activated protein kinase (MAPK) pathway components (such as *BRAF*) in melanoma, and *p53* and *EGFR* in lung cancer. Interestingly, although disruption of the same oncogenic pathways may be implicated across the three tumor types, the timing of dysregulation along the evolutionary paths can be independent.

## Discussion

We have developed a mathematical model that exploits the genetic background of each individual to characterize cancer cell heterogeneity within a tumor specimen and builds lesion hierarchies by learning from recurrent patterns across multiple patients. The approach relies on base level information from a range of next-generation sequencing data (WGS, WES and targeted sequencing) and utilizes a local optimization approach and confidence propagation steps to enable the processing of complex subclonal patterns typical of patients with advanced tumors who may have undergone multiple treatment cycles.

The local optimization approach ensures accurate tumor purity assessment across challenging samples. Our group recently implemented CLONET in precision medicine WES reports to assess adequacy of biopsy samples, highly relevant in a regulatory compliant environment. Direct assessment of CLONET performance on WES versus WGS data from 15 samples (data from [10],[17]) showed excellent agreement for *Adm.global* (Figure S8A in Additional file 10) and clonality estimates (Figure S8B in Additional file 10) where 97.6% of genes annotated as subclonal in WES are also subclonal in WGS. This approach can also be utilized to assess temporal evolution from serial samples from one patient, as observed with patient 7520, in which both his primary untreated prostate cancer and metastatic treatment-resistant tumor (biopsied three years later) were profiled. Upon correction for ploidy and global admixture, CLONET identified the gene *AURKA* as copy number neutral in the primary sample but found a gain of two copies in the late metastatic sample (Additional file 11). Amplification of *AURKA* has been described in treatment-resistant metastatic prostate cancer [29],[37], and inhibitors of AURKA are currently in clinical trials. In addition, by exploiting the local optimization approach, we were successful in following tumor dynamics across cell-free DNA samples (liquid biopsies) from castration-resistant prostate cancer patients upon deep targeted sequencing and to monitor clonal expansion and contraction of key somatic aberration from low tumor content DNA samples [38].

Here, we report the results from three common solid tumor types characterized by diverse histology and clinical course and for which we were able to detect patterns and dynamics of subclonal evolution that support the relevance of the approach. Globally, we observed great variability across and within tumor types. Targeted analyses revealed both commonalities and clear differences (Figure 6A). Notably, we identified the partial loss of chromosome 8p as a common early event in the three tumors, while the focused analysis on the long arm of chromosome 10 suggested different mechanisms of *PTEN* deregulation and participation in tumorigenesis. Finally, specific examples of subclonality, such as *MUC4* PMs in melanoma and *SOX2* gains in lung, have been identified. Understanding patterns of tumor evolution can aid in the rational development of more effective and early therapeutic approaches to directly target clonal events that are driving tumorigenesis, especially as multiple potentially actionable alterations are often identified within one tumor even at diagnosis.

It is important to note that to properly construct comprehensive tumor evolution maps, thousands of genomes are required to reach adequate statistical power when considering the range of frequencies of co-occurring aberrations. As sequencing data for multiple tumor samples and tumor types becomes accessible to the community, maps will be drafted and completed over multiple iterations. In turn, this will soon allow assigning an evolution time stamp to each new clinically profiled sample based on where the tumor genome fits into the evolution maps. Establishing a 'timeline' of cancer progression is critical for biomarkers development in the precision medicine era, allowing clinicians to more accurately gauge prognosis by adding a molecular measure of progression to standard staging and grading systems, which do not associate with molecular heterogeneity of samples (Table S3 in Additional file 5). Such an approach may allow improved clinical decision-making in a variety of cancer types, guiding the choice of management strategies and level of aggressive therapy based on how far the tumor has progressed at the genomic level.

In summary, we present a robust method that exploits next-generation sequencing data to classify somatic lesions based on their clonality within the tumor cell population. The utility of CLONET is related to transitioning next-generation sequencing efforts from the static evaluation of untreated tumor samples to the clinical arena of precision medicine where patients will be followed along a continuum of treatment modalities and a targeted therapy regimen is based on the understanding of driver mutations.

## Conclusions

Distinguishing gatekeeper or driver mutations from passenger mutations is a high priority for understanding disease progression. Knowledge of the chronology of molecular alterations can provide important insights into defining the most clinically relevant mutations that characterize important milestones in cancer. Genome sequencing of cancer samples taken during the course of precision medicine might demonstrate a wider range of genomic heterogeneity than previously observed in international genome sequencing studies. These clinical samples demonstrate more heterogeneity and admixture of both tumor and non-tumor components. To aid in unraveling the critical temporal evolution of somatic aberrations in challenging clinical tumors, we developed CLONET, a computation tool that requires only few clonal events to precisely estimate tumor purity and ploidy and then nominates the hierarchy of genomic aberrations. We demonstrate that CLONET can determine the clonality of different types of somatic aberrations, including SCNAs, PMs, and REARRs, using either WGS or WES datasets. We anticipate that with the emergence of larger genomic datasets, CLONET could help map out the evolution of molecular alterations.

## Materials and methods

### CLONET pipeline

A schematic view of the CLONET pipeline is shown in Figure S10A in Additional file 12. For this study input data were obtained as follows. Read counts of informative SNPs were extracted from BAM files using an in-house procedure, SCNAs were detected using SegSeq [39] from tumor and normal sequencing-based data, PM coordinates were as in original corresponding manuscripts, and REARRs were identified by means of dRanger and Breakpointer [14]. Finally, to avoid germline background effects, we filtered out genes (approximately 4,000) that intersect significant (size greater than 2 kb) known germline copy number variants [40].

### Fluorescence *in situ*hybridization validation of CLONET

To assess genomic deletion, disruptive translocations or polyploidy we used locus-specific dual-color FISH assays following a previously described approach [41],[42]. To assess subclonality, at least 200 nuclei per area were evaluated using a fluorescence microscope (Olympus BX51; Olympus Optical, Tokyo, Japan). The probes used for FISH assays were: SPRY2, 5′ RP11-51 N22 to 3′ RP11-478 F4; MSR1, 5′ RP11-6O24 to 3′ RP11-794E24; ERG, 5′ BAC RP11-372O17 to 3′ BAC RP11-24A11; reference probe on 10q25, BAC RP11-431P18).

### CLONET on exome and targeted sequencing data

The local approach implemented in CLONET enables the analysis of samples with few SCNAs provided that informative SNP read counts and Log R values are available. As with WGS data, individual specific informative SNPs can be identified from matched normal DNA samples. Appropriate Log R values for exome genomic segments or for each targeted area can be obtained with platform-specific strategies and provided to CLONET as input. Specifically, in the case of exome data, array-based segmented data or SCNA segments directly inferred from exome data with recent well-performing tools [43] can be utilized. CLONET combines segment input with exome-derived read counts to estimate purity and ploidy and then nominate subclonal aberrations based on sequencing data. In the case of targeted sequencing data, copy number calls derived using custom control regions and very high coverage (>1,000X) [38] allowed for CLONET-based clonality estimation even in the case of low tumor content (<10%).

### Expected distribution of the allelic fraction of a genomic segment

Consider a genomic segment that spans a set of informative SNPs for the individual of interest. For any such SNP with coverage *cov*, the total number of reads *r* supporting the reference base (reference reads) is the sum of the neutral reads (r_n_) and the active reads (r_a_) supporting the reference base. We define *β* as the ratio between neutral reads and the total number of reads spanning the SNP of interest. The probability of having *k* reference reads is then defined as the convolution of the probability of observing β*k neutral reads and (1 - *β*)*k active reads, that is:

2 $$P\left(r=k,0\beta k\beta \mathrm{cov}\right)=\mathrm{Conv}\left(P\left(r_{n}=\beta *k\right),P\left(r_{a}=\left(1\beta \beta \right)*k\right)\right)$$

We assume that *P*(*r*_*n*_ = β*k) follows a binomial distribution with number of trials equal to β*cov and probability of success equal to *ps* (that is, the probability to observe a reference read). Note that *ps* may deviate from 0.5 due to read-mapping biases [44]. All the active reads either support the reference base or the alternative base as only one allele is represented by definition of active reads. We define *N*_*ref*_ as the proportion of informative SNPs within the aberration that carry the SNP reference base in the allele represented by active reads (active allele). Then, the distribution *P*(*r*_*a*_ = (1 β*β*)*k) follows a categorical distribution with values equal to *N*_*ref*_ if *r*_*a*_ = (1 β*β*)*k and equal to (1 - *N*_*ref*_) if *r*_*a*_ = 0. Equation 2 can be written in a closed form as the sum of two binomial distributions (proof in Additional file 1):

3 $$P\left(r=k|\mathrm{cov},\beta ,N_{\mathrm{ref}},\mathrm{ps}\right)=\left(1\beta N_{\mathrm{ref}}\right)*B\left(k|\beta *\mathrm{cov},\mathrm{ps}\right)+N_{\mathrm{ref}}*B\left(k\beta \left(1\beta \beta \right)*\mathrm{cov}|\beta *\mathrm{cov},\mathrm{ps}\right)$$

where *B*(*m|n*,*p*) is the probability mass function of a binomial distribution, that is, the probability of *m* successes in *n* trials with success probability *P*.

### Estimated proportion of neutral reads for a genomic segment

The unknown values *β* and *N*_*ref*_ of Equation 3 can be inferred from the sequencing coverage at informative SNPs within the considered segment. In particular, given a segment *Seg* and a set *I* of informative SNPs within *Seg*, each informative SNP in *I* is a sample from the distribution of Equation 3. Optimization can be used to determine the values of *β* and *N*_*ref*_ for each segment. Given a random pair (*β*, *N*_*ref*_), the Kolmogorov-Smirnov nonparametric goodness-of-fit test for discrete null distribution [45] computes the likelihood that the informative SNPs in *I* are a sample from *P*(*r* = *k*|*c*,β*β*,β*N*_*ref*_,β*ps*). Next, a particle swarm optimization method [13] finds a candidate pair $\hat{\beta },\;{\hat{N}}_{\mathrm{ref}}$ that best represents the distribution of the allelic fraction of the SNPs in *I*.

### From neutral reads to non-aberrant reads

Consider a genomic segment *Seg*. If the Log R value of *Seg* supports a SCNA C, we define as aberrant those reads that cover *Seg* and are sequenced from cells harboring C. If *Seg* is a candidate somatic mono-allelic deletion, the percentage of neutral reads *β* corresponds to the percentage of reads that cover *Seg* and are sequenced from cells harboring both alleles, that is, neutral and non-aberrant reads correspond. If the Log R value of *Seg* supports a gain with integer copy number *cn* > 2, we have to re-scale *β* to obtain the percentage of sequenced cells that have copy number *cn* (that is, the percentage of non-aberrant reads)*.* For the sake of simplicity, we reason in terms of at most one copy difference between alleles. If *cn* is odd, the number of neutral reads is the sum of the neutral reads from admixed cells plus the neutral reads of the gain (Figure S10B in Additional file 12). The percentage *β*_*cn*_ of reads from cells with copy number *cn* is computed from the percentage of neutral reads *β* by removing neutral reads due to the gain, that is:

4 $$\beta_{\mathrm{cn}}=1\beta cn_{G}*\left(1\beta \beta \right)$$

If *cn* is even and at most one copy difference between alleles is allowed, then *β* is close to one, as both alleles are equally represented. This reasoning applies to any arbitrary combinations of the number of alleles (Figure S10C in Additional file 12).

### From aberrant reads to aberrant cells

Given a somatic mono-allelic deletion *M*, the local admixture *Adm.local* is defined as the proportion of cells not harboring *M* (non-aberrant cells) over the total number of cells. Let *a* define the total number of reads supporting the alternative allele (alternative reads), as the sum of neutral reads (a_n_) and active reads (a_a_) supporting the alternative base. For any informative SNP within *M*, the local admixture at *M* results:

5 $$\mathrm{Adm}.\mathrm{loca}l_{M}=\frac{\frac{r_{n}+a_{n}}{2}}{\frac{r_{n}+a_{n}}{2}+\left(r_{a}+a_{a}\right)}$$

and the proportion of non-aberrant reads covering *M* is:

6 $$\beta_{M}=\frac{r_{n}+a_{n}}{r_{n}+a_{n}+r_{a}+a_{a}}$$

By combining Equations 5 and 6, we can prove Equation 1. Additional file 1 reports a full proof of a generalized version of Equation 1 that accounts for any positive copy number.

### Uncertainty assessment and its propagation to clonality estimates

Different error sources introduce a bias into the distribution of the allelic fraction that may lead to inaccurate estimates of *β*. To optimize sensitivity and specificity, we compute the estimation uncertainty *β* around *β* and we provide a sound way to propagate *β* to clonality values. The value of *β* varies based upon the depth of sequence mean coverage (*mean.cov*) and the number of available informative SNPs (*n.info.snp*) across the segment of interest in the sample considered. The *mean.cov* controls the ability to discern the two modes of the AF distribution: the inset of Figure 2A shows that the higher *β* is, the more coverage is needed. We evaluated the uncertainty in *β* estimates as a function of these parameters by randomly generating 50 samples with given *mean.cov*, *n.info.snps*, and *β* and averaging the difference between the expected and computed *β* for a given coverage and number of SNPs. Accordingly, we generated a look-up uncertainty table for each combination of *mean.cov* and *n.info.snps* (Figure 3A).

The procedure to infer the value of *β* of a segment is independent of its Log R value. Interestingly, if we plot the Log R of a segment versus its value of *β*, segments aggregate into clusters, where each cluster corresponds to a specific copy number and to a definite clonality status (Figure S10C in Additional file 12). If we restrict our attention to putative somatic mono-allelic deletions, the cluster *B*_*min*_ with the lowest median value of *β* would likely represent 100% clonal deletions. CLONET relies on an uncertainty table (Figure 3A) to characterize *B*_*min*_. We define *B* as the set of *β* values of all the putative somatic mono-allelic deletions and *err(β)* as the uncertainty around *β*. In this context, *B*_*min*_ is defined as the smallest subset of *B* such that *min(B)* in *B*_*min*_ and for all *β* in *B* and not in *B*_*min*_, *max*(*B*_*min*_*) + err(max(B*_*min*_*)) < β’ err(β’)*. CLONET starts from *min(B)* and searches for a *β’* value such that their difference is not explained by the error table. Then, CLONET selects the median value of *B*_*min*_ as candidate *Adm.global* and computes the uncertainty around *Adm.global* as the minimum and the maximum of the β percentage of the distribution of *B*_*min*_. The value of β depends on the noise observed in the coverage (in this study we set β equal to 95%).

Given a somatic copy number *C* in a tumor sample, CLONET computes its local (*Adm.local*_*C*_) and global (*Adm.global*) admixture. The clonality *Cl*_*C*_ of *C* is then defined as the percentage of tumor cells in a sample harboring *C*:

7 $$Cl_{C}=\frac{1\beta \;\mathrm{Adm}.\mathrm{loca}l_{C}}{\;1\beta \mathrm{Adm}.\mathrm{global}}$$

The more the value of *Adm.local*_*C*_ differs from the estimated global admixture *Adm.global*, the more *C* is subclonal, namely the value of *Cl*_*C*_ approximates to 0. Finally, interval analysis [46] allows propagating uncertainty around *β* and *Adm.global* to clonality (Figure S10D in Additional file 12).

### Clonality of bi-allelic deletions

Equation 7 enables us to compute the percentage of tumor cells harboring any specific aberration (that is, its clonality) except for clonal bi-allelic deletions. For subclonal bi-allelic deletions the allelic fraction signal comes from cells with either two or one allele (Additional file 2). Consider a subclonal bi-allelic deletion where *n* (normal), *m* (mono-allelic), and *b* (bi-allelic) denote the proportion of cells with two, one, and zero alleles, respectively (*n* + *m* + *b* = 1). By Equation 1 one can assess a local estimate of the admixture (*Adm.local*) that represents the proportion of cells with two alleles in the subpopulation of cells with one or two alleles, that is *n* = Adm.local*(*n* + *m*). We can also observe that the proportion of normal cells *n* in the sample is equal to the global DNA admixture *Adm.global*. Finally, we define the clonality of a bi-allelic deletion *Cl*_*B*_ as the percentage of cells harboring a bi-allelic deletion over the number of cells with a mono- or a bi-allelic deletion, that is, *b*/(*m* + *b*). One can prove (Additional file 1) that the clonality *Cl*_*b*_ of a bi-allelic deletion is:

8 $$Cl_{b}=\frac{\mathrm{Adm}.\mathrm{global}-\mathrm{Adm}.\mathrm{local}\times \mathrm{Adm}.\mathrm{global}}{\mathrm{Adm}.\mathrm{local}\times \left(1\beta \mathrm{Adm}.\mathrm{global}\right)}$$

### Data availability

Binary sequence alignment/map (BAM) files of WGS data are accessible in the database of Genotypes and Phenotypes (dbGaP) with accession numbers phs000447.v1.p1 (prostate cancer [10]), phs000452.v1.p1 (melanoma [11]), and phs000488.v1.p1 (lung [12]); BAMs of WES data at dbGaP with accession number phs000447.v1.p1 (prostate cancer [17]).

### CLONET source code

Source code is available at [47] and at the version-controlled repository [48].

## Additional files

## Electronic supplementary material

Additional file 1: Supplementary text.(PDF 805 KB)

Additional file 2: Figure S1.: Pictorial representation of the method CLONET uses to manage bi-allelic deletions. Three types of cells are considered: normal cells (yellow) with gene A (dark brown) and gene B (light brown) present in two copies; tumor cells of type I (light red) harbor a bi-allelic deletion of both genes A and B; tumor cells of type II (dark red) have zero copies of B and one copy of A. The bottom row reports the distribution of the expected AF at informative SNPs within gene A and gene B. In pure diploid cells with two copies of genes A and B, AF is centered at 0.5. In type I tumor cells, there is no signal, as both alleles are deleted. In type II tumor cells, one allele of gene B is present and the AF assumes values 0 or 1. In a hypothetical mixture of normal and tumor cells (right panel), the distribution of AFs along gene A reports only the signal from the DNA admixture, while the distribution of gene B corresponds to a mono-allelic deletion, reflecting the fact that cells with a bi-allelic deletion do not contribute to the AF. (PDF 51 KB)

Additional file 3: Figure S2.: (A-C) Histograms of the Log R data of all the samples in the prostate (A), melanoma (B), and lung datasets (C). The left plot shows data as reported by the segmentation algorithm while the right plot shows Log R values after ploidy and *Adm.global* correction. Log R correction improves the quality of the segmentation and simplify the detection of copy number aberrations. (PDF 39 KB)

Additional file 4: Figure S3.: **(A)** Comparison between alternative allele proportions computed from WGS and MiSeq experiments. Scatterplot of the alternative allelic proportion (AP) on 18 somatic point mutations in prostate samples selected for MiSeq validation. The x-axis reports the AP observed on MiSeq data and the y-axis reports the same value computed on WGS data (Table S1 in Additional file 5). The color of a point corresponds to the clonality assigned by CLONET to the point mutation. Inset text reports Pearson product-moment correlation coefficient and associated *P*-value. **(B)** Allelic fraction (AF) of informative SNPs along the interstitial deletion between *TMPRSS2* and *ERG* and of an independent control clonal deletion for each sample reported in Figure 3A. The clonality statuses of the REARRs and of the accompanying interstitial deletions are identical. (PDF 97 KB)

Additional file 5: Table S1.: Read count of selected point mutations for MiSeq validation. Table S2. Pairwise comparison of the percentage of subclonal genomic events relative to Figure 5C. Table S3. The association between the percentage of subclonal genomic events when samples are partitioned according to patient clinical characteristics. The table shows that the clinical characteristics are not able to distinguish between less and more heterogeneous samples. (XLSX 20 KB)

Additional file 6: Figure S4.: Experimental *in situ* validation. **(A)** Low power view of adenocarcinoma Gleason score 3 + 3 = 6 in a prostatectomy specimen representative case of prostate adenocarcinoma with *SPRY2* subclonality (case STID-3042). Some areas do not have deletion of *SPRY2*, as demonstrated by the presence of two yellow signals in tumor cells by FISH (yellow box). In contrast, other areas show hemizygous deletion of *SPRY2*, as demonstrated by the presence of only one yellow signal (blue box; arrow heads) in tumor cells by FISH. **(B)** Low power view of prostate adenocarcinoma Gleason score 4 + 4 = 8 with tertiary Gleason pattern 5 in a prostatectomy specimen from a representative case of prostate adenocarcinoma with *CHD1* subclonality (case STID 2525). Some areas have homozygous deletion of *CHD1* as demonstrated by the presence of only two yellow signals (reference probe) in tumor cells by FISH (yellow box). In contrast, other areas show hemizygous deletion of *CHD1* as demonstrated by the presence of one red (*CHD1*) and two yellow signals (reference probe) in tumor cells by FISH (blue box). Note the presence of two red and two yellow signals (normal) in adjacent stromal cells, used as internal control (arrow heads). (PNG 1 MB)

Additional file 7: Figure S5.: *In silico* validation. **(A)** Scatterplot of the *Adm.global* estimates of CLONET (y-axis) versus those of ABSOLUTE (x-axis). Each dot represents a WGS melanoma sample whose color corresponds to the ploidy value estimated by ABSOLUTE. The plot shows that the ploidy of a sample does not bias the estimation. Inset text reports Pearson product-moment correlation coefficient and associated *P*-value. **(B)** Scatterplot of the *Adm.global* estimates of CLONET (y-axis) versus those of ABSOLUTE (x-axis) where each dot represents a WGS prostate sample. Inset text reports Pearson product-moment correlation coefficient and associated *P*-value. **(C)** Scatterplot of the *Adm.global* estimates of CLONET (y-axis) versus those of ABSOLUTE (x-axis) where each dot represents a WES prostate sample. Inset text reports Pearson product-moment correlation coefficient and associated *P*-value. Ploidy evaluation on the same dataset gives concordant values and found only an aneuploidy sample (case 04-1243 L). **(D)** Scatterplot of the ploidy estimates of CLONET (y-axis) versus those of ABSOLUTE (x-axis). Each dot represents a WGS melanoma sample whose color corresponds to the *Adm.global* value estimated by ABSOLUTE. The plot shows that the *Adm.global* of a sample does not bias the estimation. Inset text reports Pearson product-moment correlation coefficient and associated *P*-value. **(E)** A melanoma case (ME049T) classified as having ploidy equal to 3.05 by ABSOLUTE and equal to 1.93 by CLONET. The histogram (top) shows the Log R distribution of the segments. Yellow, violet, and orange arrows point to key Log R peaks used by both CLONET and ABSOLUTE for ploidy estimation. Beta versus Log R plot (bottom) shows the observed values for each genomic segment in sample ME049T (gray dots) and the expected position given purity and ploidy estimated by CLONET and ABSOLUTE (blue and green dots, respectively). Boxes show allele specific copy number values defined by the position in the Beta versus Log R space. (PDF 116 KB)

Additional file 8: Figure S6.: **(A)** Summary of aberrations: genomic events (GE) characterized in three tumor datasets generated through whole genome sequencing. **(B)** Histogram of the alternative allelic proportion after *Adm.global* correction of the copy number neutral somatic point mutations detected in a cohort of 264 melanoma samples from TCGA. Pie chart indicates the mean numbers of events classified as clonal (green) or subclonal (blue) across samples. **(C)** Boxplot of the percentage of clonal genes across GEs and tumor types with respect to the total number of aberrant genes. Superimposed strip-charts represent per sample data: the size of each dot is proportional to the number of genes analyzed. (PDF 60 KB)

Additional file 9: Figure S7.: Common evolution of cancer gene aberrations across tumor samples. Pairwise intersection of the tumor evolution paths of prostate, melanoma and lung samples computed on a panel of 507 cancer genes. Nodes stand for aberrant genes with the color representing the chromosome and the shape the kind of aberration. Arcs model temporal order between two aberrations found in at least three samples of the two tumor types considered. The central semicircle reports the dependencies found in the three tumor types. (PDF 31 KB)

Additional file 10: Figure S8.: Comparison of WGS- and WES-based estimates**. (A)** Scatterplot of the *Adm.global* estimates of CLONET on 15 prostate patients for which both exome data (y-axis) and WGS data (x-axis) are available. Inset text reports Pearson product-moment correlation coefficient and associated *P*-value. **(B)** Scatterplot of the percentage of clonality estimated for 23,484 genes in 15 prostate samples computed using exome data (y-axis) and WGS data (x-axis). Inset text reports Pearson product-moment correlation coefficient and associated *P*-value. (PDF 301 KB)

Additional file 11: Figure S9.: Case of tumor progression. **(A,B)** The top part of the figure shows histograms of the Log R data for a primary prostate sample (A) and a pelvic mass metastasis (B) from the same patient. **(C,D)** Upon correction for ploidy and global admixture, CLONET identifies gene *AURKA* as copy number neutral in the primary sample (C) but found a gain of two copies in the late metastatic sample (D). **(E)** The shift in the Log R values prior and after CLONET ploidy correction in the metastatic sample indicates an aneuploidy genome, as confirmed by FISH analysis that demonstrate four yellow signals (reference probe) in tumor cells. The probes that were used for FISH assays are as follows: red test probe, 3β ERG (BAC RP11-24A11); reference probe, 10q25 (BAC RP11-431P18). (PDF 132 KB)

Additional file 12: Figure S10.: CLONET method. **(A)** Schematic overview of the computing steps that lead to the definition of the tumor evolution path. **(B)** An example of a tumor specimen with two non-aberrant cells (yellow) and three aberrant cells (blue) with a duplicated genomic region (red). The *Adm.global* of this specimen is 2/5 and the percentage of aberrant reads is 4/13. Note that these values respect Equation 1. The left shows tumor cells that result from decomposing the blue aberrant cells into three normal cells and three aberrant cells with a mono-allelic deletion (brown). The percentage of neutral reads is 10/13. The value of *β* is rescaled to account for the gain by considering the proportion of aberrant reads is three times greater, that is, 1 - (3*(1 *- β*)). The bottom plot highlights that the AF of the tumor specimen and of its decomposition are the same. **(C)** Example of the distribution of the expected *β* versus Log R values in a sample with 20% of *Adm.global* and a mean ploidy of 2. Each point represents a genomic segment defined by its Log R value, computed by segmentation, and its *β* value, computed by CLONET. In particular, the blue cluster includes segments where only one allele is present in 100% of the tumor cell population (that is, they are mono-allelic clonal deletions). These segments are used to compute the *Adm.global* of the sample. The variability range of the *Adm.global* returned by CLONET considers the dispersion of the data in this cluster. **(D)** The plot shows how the variability ranges of local and global DNA admixture estimates propagate to the clonality values. Each box corresponds to a pair of local and global DNA admixture values and illustrates the clonality variability range as a function of their variability ranges. Local and global admixture variability ranges are computed from the *β* uncertainty table. (PDF 719 KB)

Below are the links to the authors’ original submitted files for images.

Authors’ original file for figure 1

Authors’ original file for figure 2

Authors’ original file for figure 3

Authors’ original file for figure 4

Authors’ original file for figure 5

Authors’ original file for figure 6

Authors’ original file for figure 7

## Abbreviations

AF: allelic fraction (fraction of reads supporting the reference base)
AP: proportion of reads supporting the alternative allele or alternative allelic proportion (fraction of reads supporting the alternative allele at a somatic point mutation position)
BAF: B allelic fraction (refer to array based data)
CLONET: CLONality Estimate in Tumors
FDR: false discovery rate
FISH: fluoresce *in situ* hybridization
PM: somatic point mutation
REARR: structural rearrangement
SCNA: somatic copy number aberration
SNP: single-nucleotide polymorphism
TCGA: The Cancer Genome Atlas
WES: whole exome sequencing
WGS: whole genome sequencing
