# Erratum to: Unraveling the clonal hierarchy of somatic genomic aberrations

**DOI:** 10.1186/s13059-017-1183-5

**Published:** 2017-05-02

**Authors:** Davide Prandi, Sylvan C. Baca, Alessandro Romanel, Christopher E. Barbieri, Juan-Miguel Mosquera, Jacqueline Fontugne, Himisha Beltran, Andrea Sboner, Levi A. Garraway, Mark A. Rubin, Francesca Demichelis

**Affiliations:** 1 Centre for Integrative Biology, University of Trento, Povo Trento, 38123 Italy; 2 The Broad Institute of MIT and Harvard, Cambridge, MA 02141 USA; 3 Dana-Farber Cancer Institute, Boston, MA 02215 USA; 4 Harvard Medical School, Boston, MA 02115 USA; 5 Department of Urology, Weill Medical College of Cornell University, New York, NY 10065 USA; 6 Department of Pathology and Laboratory Medicine, Weill Medical College of Cornell University, New York, NY 10065 USA; 7 Department of Medicine, Division of Hematology and Oncology, Weill Medical College of Cornell University, New York, NY 10065 USA; 8 Institute for Precision Medicine, Weill Medical College of Cornell University and New York Presbyterian Hospital, New York, NY 10065 USA; 9 HRH Prince Alwaleed Bin Talal Bin Abdulaziz Alsaud Institute for Computational Biomedicine, Weill Medical College of Cornell University, New York, NY 10065 USA

## Erratum

After publication of this article [1] we noticed that Figs. 1 and 2 were accidentally swapped.

The original article was updated.

The publisher apologises for these errors.
