# Supplementary material for: Unraveling the clonal hierarchy of somatic genomic aberrations
Source: Genome Biol. 2014 Aug 26;15:439. doi: 10.1186/s13059-014-0439-6 (PMC4167267; doi:10.1186/s13059-014-0439-6)
Supplement: Supplementary file 1 — Additional file 1: Supplementary text.(PDF 805 KB) [file 13059_2014_439_MOESM1_ESM.pdf]

Unraveling the clonal hierarchy of somatic genomic aberrations

Davide Prandi, Sylvan C Baca, Alessandro Romanel, Christopher E Barbieri, Juan Miguel Mosquera, Jacqueline Fontugne, Himisha Beltran, Andrea Sboner, Levi A Garraway, Mark A Rubin, Francesca Demichelis

SUPPLEMENTARY TEXT

Filtering structural rearrangements. CLONET inference engine analyzes the proportion of reads supporting each allele and infers the percentage of cells that harbors the aberrant allele. Informative SNPs fully describe the number of reads of a SCNA, while the number of reads supporting the alternative allele characterizes the clonality of a PM. In case of pair end sequencing protocols, two classes of reads represent a rearranged allele: *overlapping* single end reads that span a breakpoint, and *crossing* pair end reads, where one end maps entirely to one side and the other entirely to the other side of the breakpoint. If we consider that two genomic coordinates bp_1 and bp_2 define a canonical rearrangement, we end up with four pairs (o_1^{nA}, o_1^{A}), (c_1^{nA}, c_1^{A}), (o_2^{nA}, o_2^{A}), (c_2^{nA}, c_2^{A}), where o and c stand for the number of overlapping and crossing reads, respectively (Figure 2D), and superscripts *nA* and *A* indicates non-aberrant and aberrant reads. The value of o_1^A coincides with o_2^A, as well as c_1^A with c_2^A, because in the rearranged allele the position of bp_1 and bp_2 are joint. The ratios of $o_i^A/(o_i^A + o_i^{nA})$ and $c_i^A/(c_i^A + c_i^{nA})$ are representative of the proportion of reads supporting the rearranged allele and relate to the clonality of the aberration.

CLONET adopts strict filters to REARRs analysis. First, the breakpoint *i* in [1,2] with minimum coverage between o_1^A and o_2^A is selected to account coverage oscillations. In the attempt to be make conservative calls (minimize the number of clonal REARR called subclonal), this choice assures that the computed clonality will be the maximum among the observed values. Next, the proportions of aberrant and non-aberrant reads from overlapping and from crossing reads are compared using a proportion test between $o_i^A/(o_i^A + o_i^{nA})$ and $c_i^A/(c_i^A + c_i^{nA})$ with limit value 0.1. CLONET computes the clonality of the Rearr only in case of non-different proportions.

Classification of aberrations. To ensure robust clonality calls, we defined quality filters specific for each type of aberration. Only SCNAs with 20 or more informative SNPs with average sequence coverage of 20x (corresponding to a 5.4% uncertainty around β, Figure 3A) are considered. For PMs, we require that they are copy number neutral and that they are supported by at least 2 out of 10 reads. REARRs require special care to account for sequencing artifact near a breakpoint. Figure 2D describes how pair-end sequencing information is used to select only high confident copy number neutral REARRs. Finally, to control error propagation the clonality of an aberration is reported only if its variability range is less than 30% (Figure S10D).

For each somatic aberration, CLONET provides a direct (SCNA) or an indirect (PM, REARR) estimate of its clonality level and an associated variability range. These values are proportional to the number of cell cycles since the aberration arose and they allow assessing the history of tumor evolution. However, oscillations in the coverage, limitations in the sensitivity of the inference of β (Figure 2A) and error propagation (Figure S10D) require caution in calling clonality, especially when comparing aberrations across different tumor specimens to build the tumor evolution path. CLONET manages these factors by discretizing clonality values into four *clonality classes* (clonal, uncertain.clonal, uncertain.subclonal, and subclonal) in an aberration-dependent manner. After uncertainty propagation, a triplet $(Cl_C^{min}, Cl_C, Cl_C^{max})$ describes the clonality of a SCNA *C*, with Cl_C computed from equations (7) or (8) and (Cl_C^{min}, Cl_C^{max}) being the minimum and maximum observed clonality after uncertainty propagation. A SCNA present in less than 80% percentage of tumor cells is subclonal; uncertain is reported depending on whether or not Cl_C^{max} cross the threshold. The clonality estimate of a PM *P* derives from a single locus, but its corrected AP may assume three values $(AP_P^{min}, AP_P, AP_P^{max})$ corresponding to minimum, called, and maximum *Adm.global*, respectively. The threshold value is computed

dynamically as the 95% confidence limit of the expected distribution of the number of reads supporting alternative base. The same strategy applies to REARRs, with the alternative allele represented by the re-fished reads around the breakpoints.

Aneuploidy. Aneuploidy is a pathological condition in which the mean number of alleles per chromosome differs from the number characteristic of a specific species. Since the number of reads covering a given genomic position is proportional to the number of alleles, aneuploidy will affect the mean coverage of a sample. Let's define the Log R of a genomic position *P*, as the logarithm base 2 of the ratio between tumor and normal coverage at *P*, normalized by the mean tumor and normal coverage ratio. Implicitly here we assume that the mean tumor coverage corresponds to the coverage of a diploid genome and aneuploidy will shift the Log R signal as observed for metastatic treatment resistant prostate tumor 7520 (Figure S9B and S9D). Therefore, different ploidy estimations can lead to dramatically different SCNA calls. Figure S5E shows a melanoma case ME049T from Berger et al [1] originally classified as ploidy equal to 3.05 by ABSOLUTE [2] and equal to 1.93 by CLONET. As a consequence, segments with Log R value near 0 can be interpreted as 4 (ABSOLUTE) or 2 (CLONET) copies. To improve the sensitivity, CLONET expands Log R data by adding information on allelic fraction imbalance at informative SNPs (i.e., Beta) (Figure S5E, bottom) and try to fit observed and expected data. Mathematical details follow.

Let's consider a sample comprising a collection of aberrant and non-aberrant diploid cells. We define cn_A and cn_B as the number of copies of alleles *A* and *B* in each aberrant cell. The allelic fraction of an aberrant cell with arbitrary allelic copy numbers cn_A and cn_B is equivalent to the allelic fraction of a population including $2 * min(cn_A, cn_B)$ cells with both allele *A* and *B*, and $|cn_A - cn_B|$ cells with only one allele (i.e., with a dummy mono-allelic deletion) (Figure S10B). Therefore, the apparent local admixture is the sum of the percentage of normal

cells in the collection (*Adm.global*) plus the percentage of apparent tumor cells with two alleles (e.g., in Figure S10B right panel, this correspond to 3/6):

$$Adm.local.apparent = Adm.global + (1 - Adm.global)\frac{2 * min(cn_A, cn_B)}{2 * min(cn_A, cn_B) + |cn_A - cn_B|} \quad (1)$$

Since we are in case of a (dummy) mono-allelic deletion, we can apply equation (1) and obtain the expected apparent percentage of reads from non-aberrant cells

$$\beta.apparent = \frac{2 * Adm.local.apparent}{1 + Adm.local.apparent} \quad (2)$$

Interestingly, this value corresponds to the percentage of neutral reads *β* (Figure S10B). Given cn_A, cn_B, and the corrected value of β_{cn}, we can also compute the expected Log R for each genomic segment. By definition, the total copy number of each aberrant cell (cn_{Aber}) is equal to cn_A+cn_B. If we assume that the copy number of non-aberrant cells is 2, we can describes the observed ratio between the tumor and the normal signal, normalized by the mean tumor and normal ploidy, as

$$\log R = \log_2 \frac{\frac{\beta_{cn} * 2 + (1 - \beta_{cn}) * cn_{Ab}}{2}}{\frac{Ploidy}{2}} \quad (3)$$

where the numerator is the sum of the admixture ($\beta_{cn} * 2$) and of the aberrant ($(1 - \beta_{cn}) * cn_{Ab}$) components of the cell collection. The denominator of equation (3) accounts for (possible) normalization of the signal.

Equations (2) and (3) completely define the *β vs* Log R space reported in Figure 2c and Figure S10C. In principle, we can use the *β vs* Log R space to find a combination of *Adm.global* and *Ploidy* that best fits the observed data, but, for highly heterogeneous samples (subclonality) we cannot *a priori* exclude any combination of subclonal copy number signal. However, when cn_A is equal to cn_B, *β* is always equal to 1 and it is invariant to both *Adm.global* and *Ploidy*. CLONET searches for clusters of *β* values close to 1 and adjusts Log R values in such a way that new values correspond to cn_A = cn_B, namely 0, 1, 1.58, 2, etc. In case of ambiguity

(different local minima of RMSE between adjusted and expected Log R values) the system flags the sample as unresolved.

PROOFS

Proof of Equation 3 (Allelic Fraction Distribution)

$P(r = k, 0 \leq k \leq c) =$

$Conv\big(P(r_{nA} = \beta k), P(r_A = (1-\beta)k)\big) =$ (by definition of convolution)

$\sum_{i=0}^{c} P(r_{nA} = k - i) * P(r_A = i) =$ (as $P(r_A = i)$ is defined for $i = 0$ or $i = (1-\beta) * c$)

$P(r_{nA} = k) * \big(1 - N_{ref}\big) + P(r_{nA} = k - (1-\beta)c) * N_{ref} =$

$B(k|\beta c, ps) * \big(1 - N_{ref}\big) + B(k - (1-\beta)c)|\beta c, ps) * N_{ref}$

Proof of Equation 1 (Relation between Adm.local and β)

Given a SCNA (aberration) *C*, its local DNA admixture is defined as the percentage of non-aberrant cells over the total number of cells. We overload symbols nA and A to indicate both non-aberrant and aberrant cells and their number when unambiguous. Then we can write the following

$$Adm.local_C = \frac{nA}{nA + A}$$

An unbiased sequencing experiment implies that the number of cells in a specimen is proportional to the coverage divided by the number of alleles (integer copy number). Therefore, given a genomic position within *C*, nA is proportional to the number of non-aberrant reads c_{nA} divided by the integer copy number cn_{nA} of non-aberrant cells. The same apply to aberrant cells where c_A is the number of aberrant reads and cn_A is the integer copy number of aberrant cells. For any arbitrary genomic position in *C*, the local admixture is defined as

$$Adm.local_C = \frac{\frac{c_{nA}}{cn_{nA}}}{\frac{c_{nA}}{cn_{nA}} + \frac{c_A}{cn_A}}$$

By focusing to informative SNPs and decomposing the coverage as the sum of reference and alternative reads, we can write

$$Adm.local_C = \frac{\frac{r_{nA}+a_{nA}}{cn_{nA}}}{\frac{r_{nA}+a_{nA}}{cn_{nA}} + \frac{r_A+a_A}{cn_A}}$$

Interestingly, the equation describing the local admixture estimate in an informative SNP position is similar to the one describing the percentage of non-aberrant reads β_C in C

$$\beta_C = \frac{r_{nA} + a_{nA}}{r_{nA} + a_{nA} + r_A + a_A}$$

In the following we will prove that for cn_A and cn_{nA} greater that zero there is a precise relation between $Adm.local_C$ and β_C.

$$Adm.local_C = \frac{\frac{r_{nA}+a_{nA}}{cn_{nA}}}{\frac{r_{nA}+a_{nA}}{cn_{nA}} + \frac{r_A+a_A}{cn_A}} =$$

$$\frac{\frac{r_{nA}+a_{nA}}{cn_{nA}}}{\frac{cn_A\, r_{nA}+cn_A\, a_{nA}+cn_{nA}\, r_A+cn_{nA}\, a_A}{cn_{nA}\, cn_A}} =$$

$$\frac{\frac{cn_A\, r_{nA}+cn_A\, a_{nA}}{cn_N\, cn_T}}{\frac{cn_A\, r_{nA}+cn_A\, a_{nA}+cn_{nA}\, r_A+cn_{nA}\, a_A}{cn_{nA}\, cn_A}} =$$

$$\frac{cn_A\, r_{nA} + cn_A\, a_{nA}}{cn_A\, r_{nA} + cn_A\, a_{nA} + cn_{nA}\, r_A + cn_{nA}\, a_A} =$$

$$\frac{cn_A\, r_{nA} + cn_A\, a_{nA}}{cn_A\, r_{nA} + cn_A\, a_{nA} + cn_{nA}\, r_A + cn_{nA}\, a_A - cn_{nA}\, r_{nA} - cn_{nA}\, a_{nA} + cn_{nA}\, r_{nA} + cn_{nA}\, a_{nA}} =$$

$$\frac{cn_A\, r_{nA} + cn_A\, a_{nA}}{(cn_A - cn_{nA})(r_{nA} + a_{nA}) + cn_{nA}\, r_A + cn_{nA}\, a_A + cn_{nA}\, r_{nA} + cn_{nA}\, a_{nA}} =$$

$$\frac{cn_A\, r_{nA} + cn_A\, a_{nA}}{(cn_A - cn_{nA})(r_{nA} + a_{nA}) + cn_{nA}(r_A + a_A + r_{nA} + a_{nA})} =$$

$$\frac{\frac{cn_A\, r_{nA}+cn_A\, a_{nA}}{r_A+a_A+r_{nA}+a_{nA}}}{\frac{(cn_A-cn_{nA})(r_{nA}+a_{nA})+cn_{nA}(r_A+a_A+r_{nA}+a_{nA})}{r_A+a_A+r_{nA}+a_{nA}}} =$$

$$\frac{cn_A \frac{r_{nA}+a_{nA}}{r_A+a_A+r_{nA}+a_{nA}}}{(cn_A - cn_{nA})\frac{r_{nA}+a_{nA}}{r_A+a_A+r_T+a_T} + cn_{nA}\frac{r_A+a_A+r_{nA}+a_{nA}}{r_A+a_A+r_{nA}+a_{nA}}} =$$

$$\frac{cn_A \beta}{(cn_A - cn_{nA})\beta + cn_A}$$

Proof of Equation 8 (Monoallelic Deletion)

Let's consider a subclonal bi-allelic deletion where *n* (normal), *m* (mono-allelic), and *b* (bi-allelic) denote the proportion of cells with two, one, and zero alleles, respectively (*n*+*m*+*b*=1). By definition we have

$$1 - Cl_b = \frac{m}{m+b} = \frac{m}{m+1-m-n} = \frac{m}{1-n}$$

We observe that *n=Adm.local*(n+m)* and that *n=Adm.global* and therefore

$$m = \frac{Adm.global - Adml.local * Adm.global}{Adm.local}$$

We can the conclude that

$$1 - Cl_b = \frac{\frac{Adm.global - Adml.local * Adm.global}{Adm.local}}{1 - Adm.global} = \frac{Adm.global - Adml.local * Adm.global}{Adm.local * (1 - Adm.global)}$$

SUPPLEMENTARY TABLES AND FIGURES LEGENDS

Supplementary Table S1. Read count of selected point mutations for MiSeq validation.

Supplementary Table S2. Pairwise comparison of the percentage of subclonal genomic events relative to Figure 5C.

Supplementary Table S3. The table reports the association between the percentage of subclonal genomic events when samples are partitioned accordingly with patients' clinical characteristics. The table shows that the clinical characteristics are not able to distinguish between less and more heterogeneous samples.

Supplementary Figure S1.
Pictorial representation of the method CLONET uses to manage bi-allelic deletions. Three types of cells are considered: normal cells (yellow) with gene A (dark brown) and gene B (light brown) present in two copies; tumor cells of type I (light red) harbor a bi-allelic deletion of both genes A and B; tumor cells of type II (dark red) have zero copies of B and one copy of A. The bottom row reports the distribution of the expected AF at informative SNPs within gene A and gene B. In pure diploid cells with two copies of genes A and B, AF is centered in 0.5. In type I tumor cells, there is no signal, as both alleles are deleted. In type II tumor cells, one allele of gene B is present and the AF assumes values 0 or 1. In a hypothetical mixture of normal and tumor cells (right panel), the distribution of AFs along gene A reports only the signal from the DNA admixture, while the distribution of gene B corresponds to a mono-allelic deletion, reflecting the fact that cells with a bi-allelic deletion do not contribute to the AF.

Supplementary Figure S2.
(A-C) Histogram of the Log R data of all the samples in the prostate, melanoma, and lung dataset, respectively. The left plot shows data as reported by the segmentation algorithm while the right plot shows Log R values after ploidy and *Adm.global* correction. Log R correction improves the quality of the segmentation and simplify the detection of copy number aberrations.

Supplementary Figure S3.
(A) Comparison between alternative allele proportions computed from WGS and MiSeq experiments. Scatterplot of the alternative allelic proportion (AP) on 18 somatic point mutations

in prostate samples selected for MiSeq validation. The x axis reports the AP observed on MiSeq data and the y axis reports the same value computed on WGS data (Supplementary Table S1). The color of a point corresponds to the clonality assigned by CLONET to the point mutation. Inset text reports Pearson product-moment correlation coefficient and associated p-value. **(B)** Allelic fraction (AF) of informative SNPs along the interstitial deletion between *TMPRSS2* and *ERG* and of an independent control clonal deletion for each sample is reported in Fig. 3A. The clonality statuses of the Rearrs and of the accompanying interstitial deletions are identical.

Supplementary Figure S4. Experimental in situ validation.

(A) Low power view of adenocarcinoma Gleason score 3+3=6 in a prostatectomy specimen representative case of prostate adenocarcinoma with *SPRY2* subclonality (Case STID-3042). **(**yellow box) Some areas do not have deletion of *SPRY2* as demonstrated by the presence of two yellow signals in tumor cells by FISH. (blue box) In contrast, other areas show hemizygous deletion of *SPRY2* as demonstrated by the presence of only one yellow signal (arrow heads) in tumor cells by FISH. **(B)** Low power view of prostate adenocarcinoma Gleason score 4+4=8 with tertiary Gleason pattern 5, in a prostatectomy specimen representative case of prostate adenocarcinoma with *CHD1* subclonality (Case STID 2525). (yellow box) Some areas have homozygous deletion of *CHD1* as demonstrated by the presence of only two yellow signals (reference probe) in tumor cells by FISH. (blue box) In contrast, other areas show hemizygous deletion of *CHD1* as demonstrated by the presence of one red *(CHD1)* and two yellow signals (reference probe) in tumor cells by FISH. Note the presence of two red and two yellow signals (normal) in adjacent stromal cells, used as internal control (arrow heads).

Supplementary Figure S5. In silico validation.

(A) Scatterplot of the *Adm.global* estimates of CLONET (y axis) vs ABSOLUTE (x axis). Each dot represents a WGS melanoma sample whose color corresponds to the *ploidy* value estimated by ABSOLUTE. The plot shows that the *ploidy* of a sample does not bias the estimation. Inset text reports Pearson product-moment correlation coefficient and associated p-value. **(B)** Scatterplot of the *Adm.global* estimates of CLONET (y axis) vs ABSOLUTE (x axis) where each dot represents a WGS prostate sample. Inset text reports Pearson product-moment correlation coefficient and associated p-value. **(C)** Scatterplot of the Adm.global estimates of CLONET (y axis) vs ABSOLUTE (x axis) where each dot represents a WES prostate sample. Inset text reports Pearson product-moment correlation coefficient and associated p-value. Ploidy evaluation on the same dataset gives concordant values and found only an aneuploidy

sample (case 04-1243L). **(D)** Scatterplot of the *ploidy* estimates of CLONET (y axis) vs ABSOLUTE (x axis). Each dot represents a WGS melanoma sample whose color corresponds to the *Adm.global* value estimated by ABSOLUTE. The plot shows that the *Adm.global* of a sample does not bias the estimation. Inset text reports Pearson product-moment correlation coefficient and associated p-value. **(E)** A melanoma case (ME049T) classified as having ploidy equal to 3.05 by ABSOLUTE and equal to 1.93 by CLONET. The histogram (top) shows the Log R distribution of the segments. Yellow, violet, and orange arrows point to key Log R peaks used by both CLONET and ABSOLUTE for ploidy estimation. Beta vs Log R plot (bottom) shows the observed values for each genomic segment in sample ME049T (gray dots) and the expected position given purity and ploidy estimated by CLONET and ABSOLUTE (blue and green dots, respectively). Boxes show allele specific copy number values defined by the position in the Beta vs Log R space.

Supplementary Figure S6

(A) Summary of aberrations: Genomic Events (GE) characterized in three tumor datasets generated through whole genome sequencing. **(B)** Histogram of the alternative allelic proportion after *Adm.global* correction of the copy number neutral somatic point mutations detected in a cohort of 264 melanoma samples from TCGA. Pie chart indicates the mean numbers of events classified as clonal (green) or subclonal (blue) across samples. **(C)** Boxplot of the percentage of clonal genes across GEs and tumor types with respect to the total number of aberrant genes. Superimposed strip-charts represent per sample data: the size of each dot is proportional to the number of genes analyzed.

Supplementary Figure S7. Common evolution of cancer gene aberrations across tumor samples.

Pairwise intersection of the tumor evolution paths of prostate, melanoma and lung samples computed on a panel of 507 cancer genes. Nodes stand for aberrant genes with the color representing the chromosome and the shape the kind of aberration. Arcs model temporal order between two aberrations found in at least three samples of the two tumor types considered. The central semicircle reports the dependencies found in the three tumor types.

Supplementary Figure S8. Comparison of WGS and WES based estimates**.**

(A) Scatterplot of the *Adm.global* estimates of CLONET on 15 prostate patients for which both exome data (y axis) and WGS data (x axis) are available. Inset text reports Pearson product-

moment correlation coefficient and associated p-value. **(B)** Scatterplot of the percentage of clonality estimated for 23484 genes in 15 prostate samples computed using exome data (y axis) and WGS data (x axis). Inset text reports Pearson product-moment correlation coefficient and associated p-value.

Supplementary Figure S9. Case of tumor progression.

The top part of the figure shows the histograms of the Log R data for a primary prostate sample **(A)** and a pelvic mass metastasis **(B)** from the same patient. Upon correction for ploidy and global admixture, CLONET identifies gene *AURKA* as copy number neutral in the primary sample **(C)** but found a gain of 2 copies in the late metastatic sample **(D)**. The shift in the Log R values prior and after CLONET ploidy correction in the metastatic sample indicates an aneuploidy genome, as confirmed by FISH analysis that demonstrate four yellow signals (reference probe) in tumor cells **(E)**. The probes that were used for FISH assays are as follows: red test probe - 3' ERG (BAC RP11-24A11); reference probe - 10q25 (BAC RP11-431P18).

Supplementary Figure S10. CLONET method.

(A) Schematic overview of the computing steps that lead to the definition of the tumor evolution path. **(B)** an example of tumor specimen with 2 non-aberrant cells (yellow) and 3 aberrant cells (blue) with a duplicated genomic region (red). The *Adm.global* of this specimen is 2/5 and the percentage of aberrant reads is 4/13. Note that these values respect Equation (1). The left shows tumor cells that results decomposing the blue aberrant cells into 3 normal cells and 3 aberrant cells with a mono-allelic deletion (brown). The percentage of neutral reads is 10/13. The value of β is rescaled to account for the gain by considering the proportion of aberrant reads is three times greater, that is, $1\text{-}(3*(1\text{-}\ \beta))$. The bottom plot highlights that the AF of the tumor specimen and of its decomposition are the same. **(C)** example of the distribution of the expected β vs Log R values in a sample with 20% of *Adm.global* and a mean ploidy of 2. Each point represents a genomic segment defined by its Log R value, computed by segmentation, and its β value, computed by CLONET. In particular, the blue cluster includes segments where only one allele is present in the 100% of the tumor cells population (i.e., they are mono-allelic clonal deletions). These segments are used to compute the *Adm.global* of the sample. The variability range of the *Adm.global* returned by CLONET considers the dispersion of the data in this cluster. **(D)** the plot shows how the variability ranges of local and global DNA admixture estimates propagate to the clonality values. Each box corresponds to a pair of local and global DNA admixture values and illustrates the clonality variability range as a function of their

variability ranges. Local and global admixture variability ranges are computed from β uncertainty table.

References

1. Berger MF, Hodis E, Heffernan TP, Deribe YL, Lawrence MS, Protopopov A, Ivanova E, Watson IR, Nickerson E, Ghosh P, et al: **Melanoma genome sequencing reveals frequent PREX2 mutations.** *Nature* 2012, **485:**502-506.
2. Carter SL, Cibulskis K, Helman E, McKenna A, Shen H, Zack T, Laird PW, Onofrio RC, Winckler W, Weir BA, et al: **Absolute quantification of somatic DNA alterations in human cancer.** *Nat Biotechnol* 2012, **30:**413-421.
