# Supplementary material for: Unraveling the clonal hierarchy of somatic genomic aberrations
Source: Genome Biol. 2014 Aug 26;15:439. doi: 10.1186/s13059-014-0439-6 (PMC4167267; doi:10.1186/s13059-014-0439-6)

Supplementary Figure 1

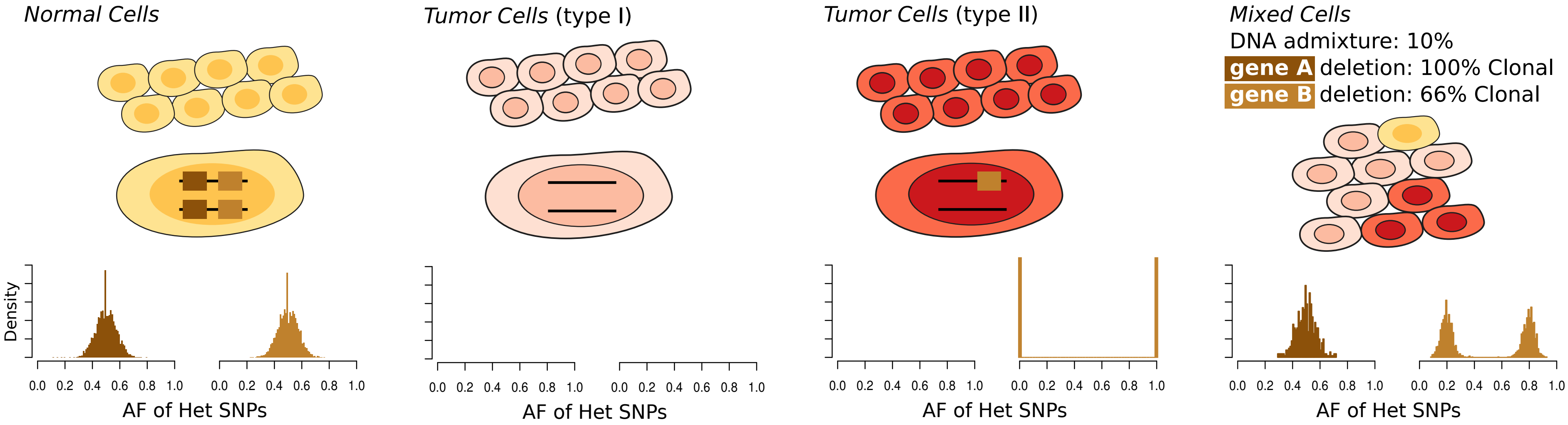

Supplement: Supplementary file 2 — Additional file 2: Figure S1.: Pictorial representation of the method CLONET uses to manage bi-allelic deletions. Three types of cells are considered: normal cells (yellow) with gene A (dark brown) and gene B (light brown) present in two copies; tumor cells of type I (light red) harbor a bi-allelic deletion of both genes A and B; tumor cells of type II (dark red) have zero copies of B and one copy of A. The bottom row reports the distribution of the expected AF at informative SNPs within gene A and gene B. In pure diploid cells with two copies of genes A and B, AF is centered at 0.5. In type I tumor cells, there is no signal, as both alleles are deleted. In type II tumor cells, one allele of gene B is present and the AF assumes values 0 or 1. In a hypothetical mixture of normal and tumor cells (right panel), the distribution of AFs along gene A reports only the signal from the DNA admixture, while the distribution of gene B corresponds to a mono-allelic deletion, reflecting the fact that cells with a bi-allelic deletion do not contribute to the AF. (PDF 51 KB) [file 13059_2014_439_MOESM2_ESM.pdf]
