# Supplementary figures and images for: Unraveling the clonal hierarchy of somatic genomic aberrations
Source: Genome Biol. 2014 Aug 26;15:439. doi: 10.1186/s13059-014-0439-6 (PMC4167267; doi:10.1186/s13059-014-0439-6)

## Supplementary Figure 2

A

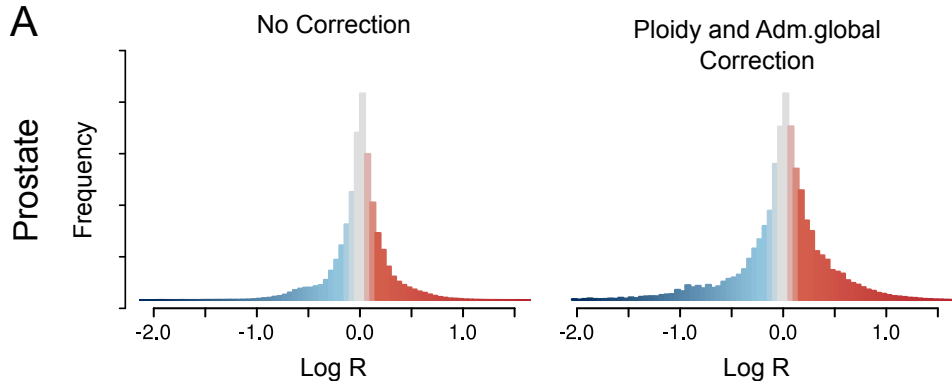

B

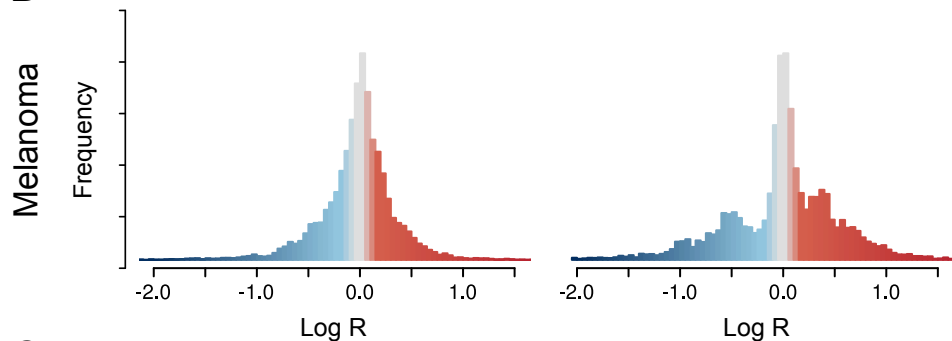

C

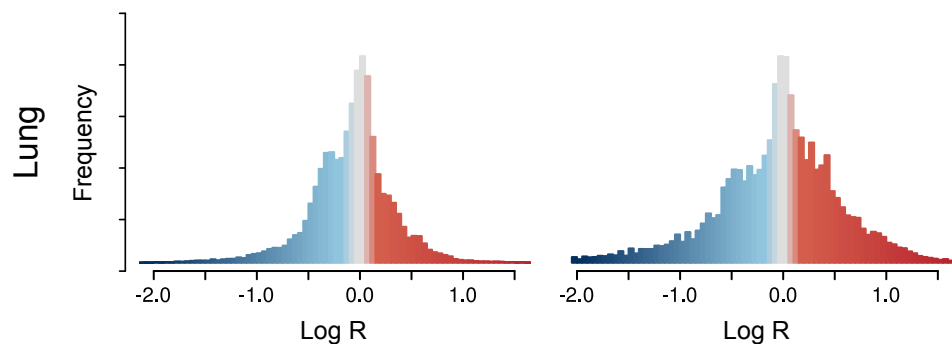

Supplement: Supplementary file 3 — Additional file 3: Figure S2.: (A-C) Histograms of the Log R data of all the samples in the prostate (A), melanoma (B), and lung datasets (C). The left plot shows data as reported by the segmentation algorithm while the right plot shows Log R values after ploidy and Adm.global correction. Log R correction improves the quality of the segmentation and simplify the detection of copy number aberrations. (PDF 39 KB) [file 13059_2014_439_MOESM3_ESM.pdf]

# Supplementary Figure 3

**A**

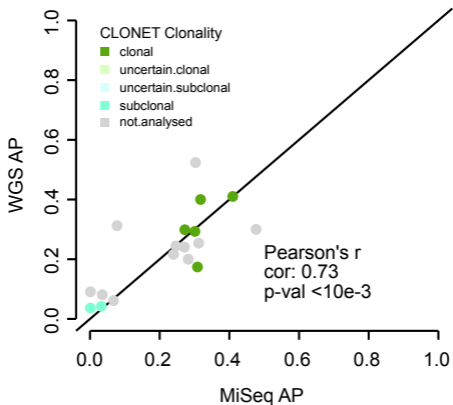

**B**

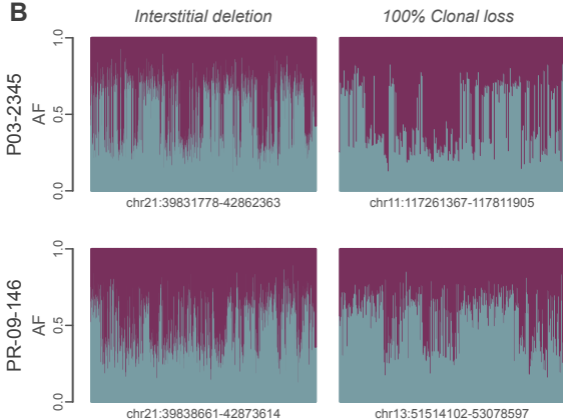

Supplement: Supplementary file 4 — Additional file 4: Figure S3.: (A) Comparison between alternative allele proportions computed from WGS and MiSeq experiments. Scatterplot of the alternative allelic proportion (AP) on 18 somatic point mutations in prostate samples selected for MiSeq validation. The x-axis reports the AP observed on MiSeq data and the y-axis reports the same value computed on WGS data (Table S1 in Additional file 5). The color of a point corresponds to the clonality assigned by CLONET to the point mutation. Inset text reports Pearson product-moment correlation coefficient and associated P-value. (B) Allelic fraction (AF) of informative SNPs along the interstitial deletion between TMPRSS2 and ERG and of an independent control clonal deletion for each sample reported in Figure 3A. The clonality statuses of the REARRs and of the accompanying interstitial deletions are identical. (PDF 97 KB) [file 13059_2014_439_MOESM4_ESM.pdf]

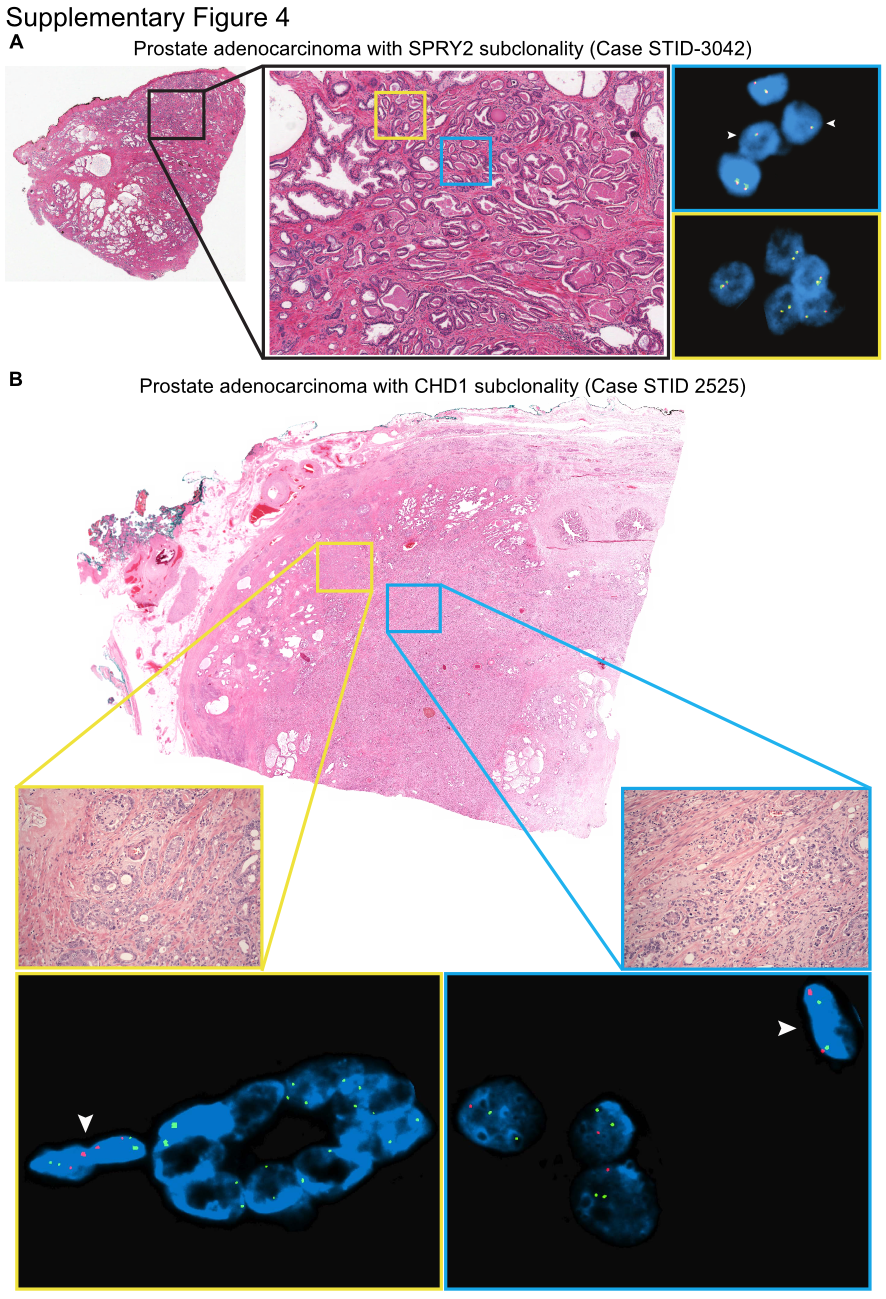

Supplement: Supplementary file 6 — Additional file 6: Figure S4.: Experimental in situ validation. (A) Low power view of adenocarcinoma Gleason score 3 + 3 = 6 in a prostatectomy specimen representative case of prostate adenocarcinoma with SPRY2 subclonality (case STID-3042). Some areas do not have deletion of SPRY2, as demonstrated by the presence of two yellow signals in tumor cells by FISH (yellow box). In contrast, other areas show hemizygous deletion of SPRY2, as demonstrated by the presence of only one yellow signal (blue box; arrow heads) in tumor cells by FISH. (B) Low power view of prostate adenocarcinoma Gleason score 4 + 4 = 8 with tertiary Gleason pattern 5 in a prostatectomy specimen from a representative case of prostate adenocarcinoma with CHD1 subclonality (case STID 2525). Some areas have homozygous deletion of CHD1 as demonstrated by the presence of only two yellow signals (reference probe) in tumor cells by FISH (yellow box). In contrast, other areas show hemizygous deletion of CHD1 as demonstrated by the presence of one red (CHD1) and two yellow signals (reference probe) in tumor cells by FISH (blue box). Note the presence of two red and two yellow signals (normal) in adjacent stromal cells, used as internal control (arrow heads). (PNG 1 MB) [file 13059_2014_439_MOESM6_ESM.png]

Supplementary Figure 5

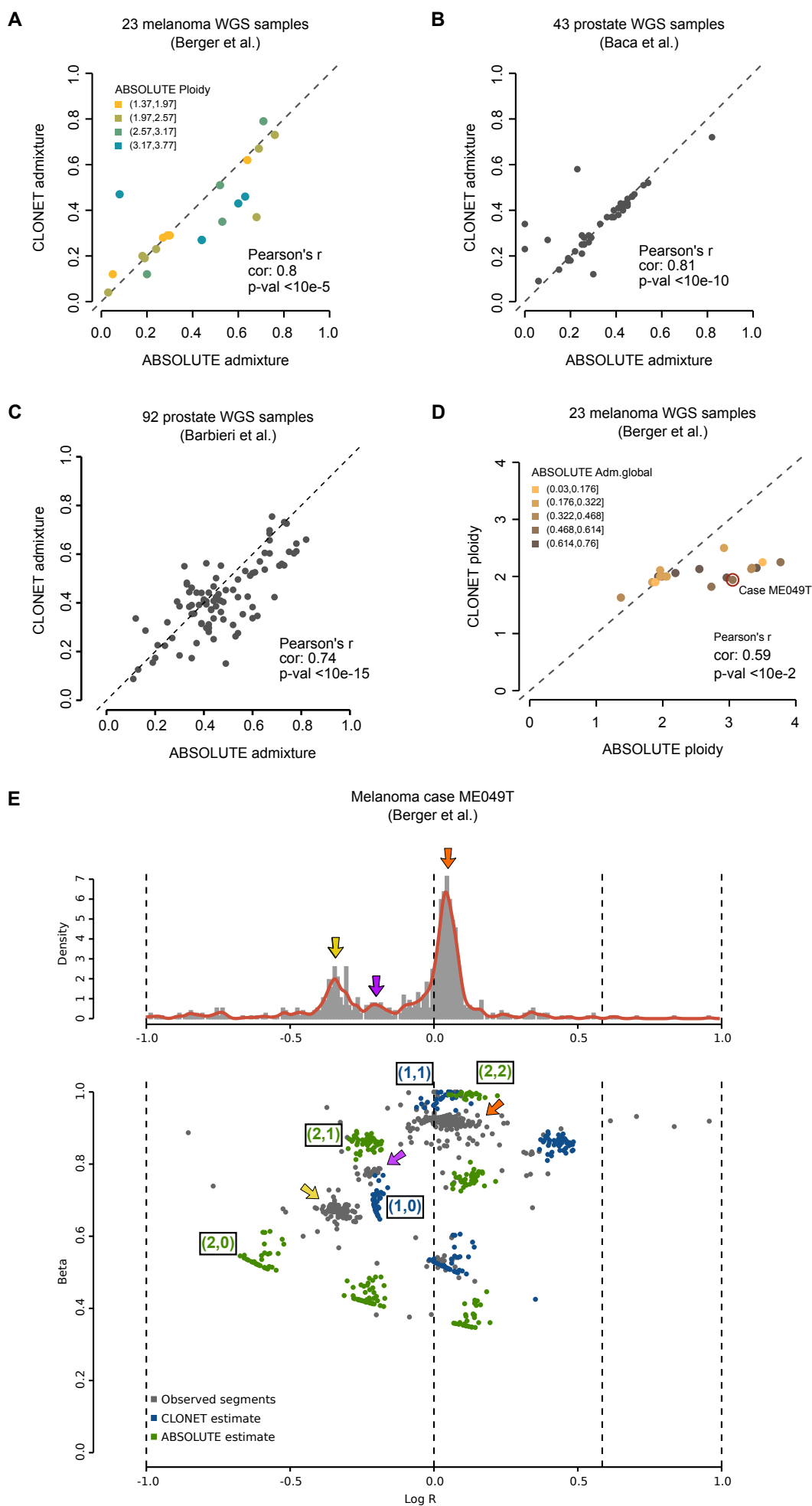

Supplement: Supplementary file 7 — Additional file 7: Figure S5.: In silico validation. (A) Scatterplot of the Adm.global estimates of CLONET (y-axis) versus those of ABSOLUTE (x-axis). Each dot represents a WGS melanoma sample whose color corresponds to the ploidy value estimated by ABSOLUTE. The plot shows that the ploidy of a sample does not bias the estimation. Inset text reports Pearson product-moment correlation coefficient and associated P-value. (B) Scatterplot of the Adm.global estimates of CLONET (y-axis) versus those of ABSOLUTE (x-axis) where each dot represents a WGS prostate sample. Inset text reports Pearson product-moment correlation coefficient and associated P-value. (C) Scatterplot of the Adm.global estimates of CLONET (y-axis) versus those of ABSOLUTE (x-axis) where each dot represents a WES prostate sample. Inset text reports Pearson product-moment correlation coefficient and associated P-value. Ploidy evaluation on the same dataset gives concordant values and found only an aneuploidy sample (case 04-1243 L). (D) Scatterplot of the ploidy estimates of CLONET (y-axis) versus those of ABSOLUTE (x-axis). Each dot represents a WGS melanoma sample whose color corresponds to the Adm.global value estimated by ABSOLUTE. The plot shows that the Adm.global of a sample does not bias the estimation. Inset text reports Pearson product-moment correlation coefficient and associated P-value. (E) A melanoma case (ME049T) classified as having ploidy equal to 3.05 by ABSOLUTE and equal to 1.93 by CLONET. The histogram (top) shows the Log R distribution of the segments. Yellow, violet, and orange arrows point to key Log R peaks used by both CLONET and ABSOLUTE for ploidy estimation. Beta versus Log R plot (bottom) shows the observed values for each genomic segment in sample ME049T (gray dots) and the expected position given purity and ploidy estimated by CLONET and ABSOLUTE (blue and green dots, respectively). Boxes show allele specific copy number values defined by the position in the Beta versus L [file 13059_2014_439_MOESM7_ESM.pdf]

Figure S6

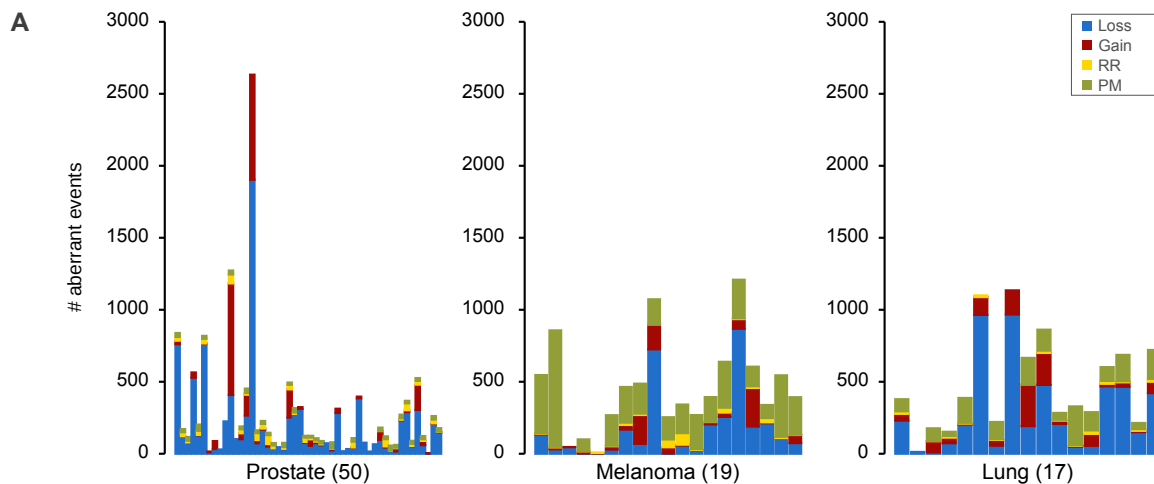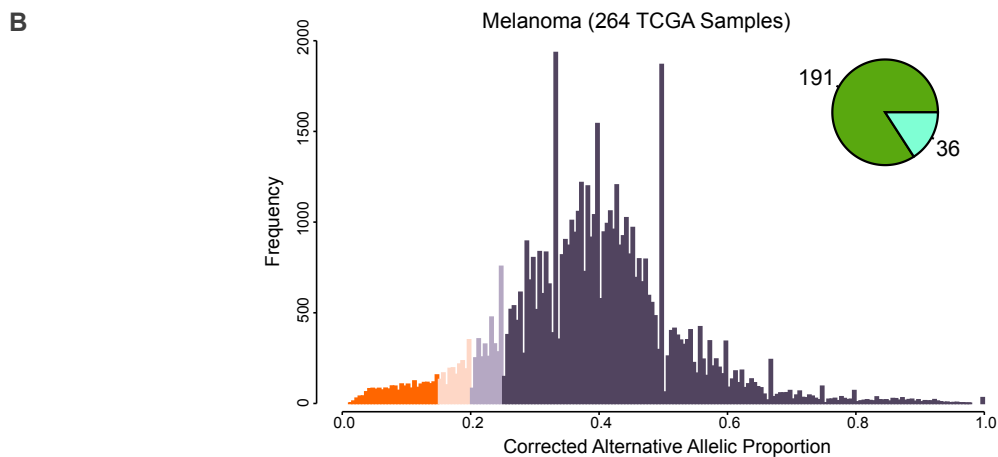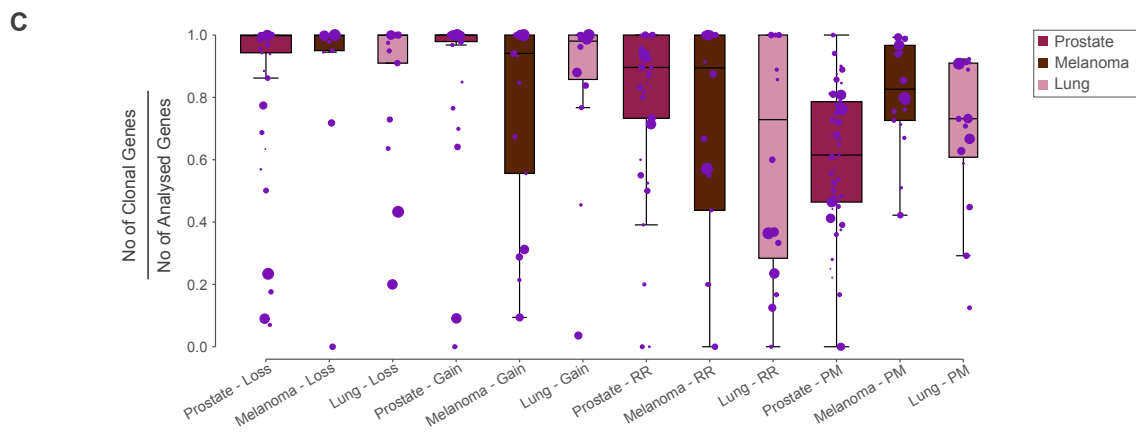

Supplement: Supplementary file 8 — Additional file 8: Figure S6.: (A) Summary of aberrations: genomic events (GE) characterized in three tumor datasets generated through whole genome sequencing. (B) Histogram of the alternative allelic proportion after Adm.global correction of the copy number neutral somatic point mutations detected in a cohort of 264 melanoma samples from TCGA. Pie chart indicates the mean numbers of events classified as clonal (green) or subclonal (blue) across samples. (C) Boxplot of the percentage of clonal genes across GEs and tumor types with respect to the total number of aberrant genes. Superimposed strip-charts represent per sample data: the size of each dot is proportional to the number of genes analyzed. (PDF 60 KB) [file 13059_2014_439_MOESM8_ESM.pdf]

Supplementary Figure 7

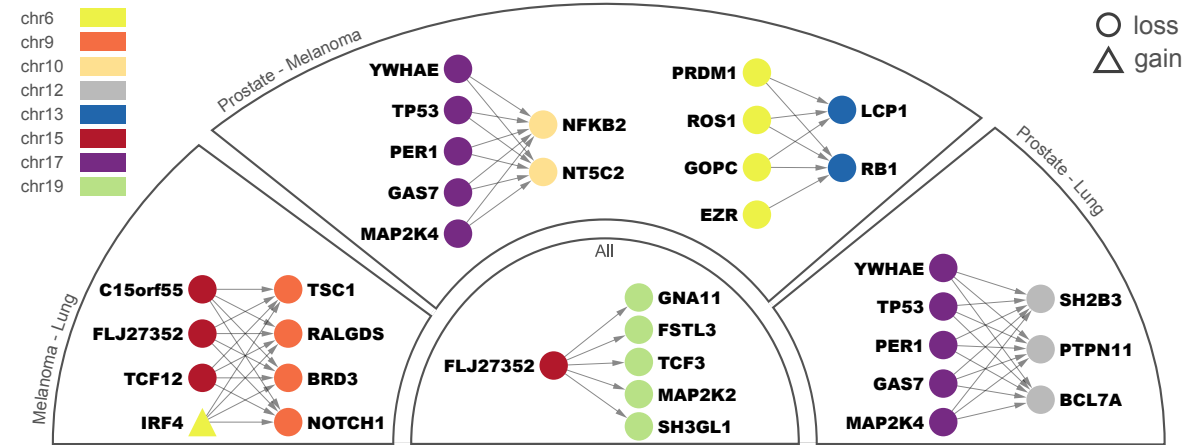

Supplement: Supplementary file 9 — Additional file 9: Figure S7.: Common evolution of cancer gene aberrations across tumor samples. Pairwise intersection of the tumor evolution paths of prostate, melanoma and lung samples computed on a panel of 507 cancer genes. Nodes stand for aberrant genes with the color representing the chromosome and the shape the kind of aberration. Arcs model temporal order between two aberrations found in at least three samples of the two tumor types considered. The central semicircle reports the dependencies found in the three tumor types. (PDF 31 KB) [file 13059_2014_439_MOESM9_ESM.pdf]

Supplementary Figure 8

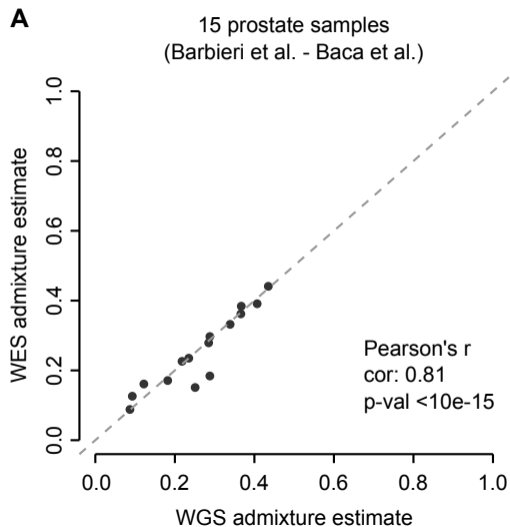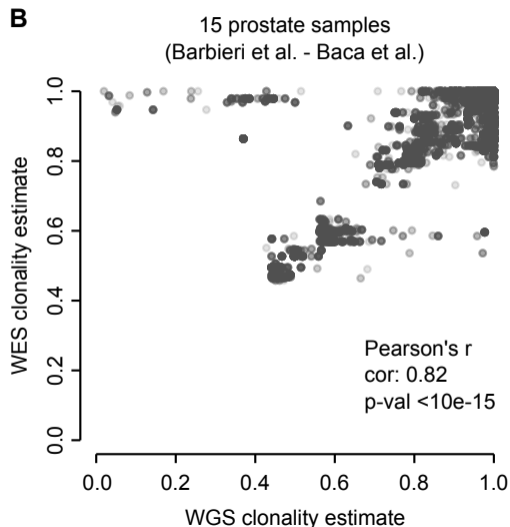

Supplement: Supplementary file 10 — Additional file 10: Figure S8.: Comparison of WGS- and WES-based estimates. (A) Scatterplot of the Adm.global estimates of CLONET on 15 prostate patients for which both exome data (y-axis) and WGS data (x-axis) are available. Inset text reports Pearson product-moment correlation coefficient and associated P-value. (B) Scatterplot of the percentage of clonality estimated for 23,484 genes in 15 prostate samples computed using exome data (y-axis) and WGS data (x-axis). Inset text reports Pearson product-moment correlation coefficient and associated P-value. (PDF 301 KB) [file 13059_2014_439_MOESM10_ESM.pdf]

Patient 7520

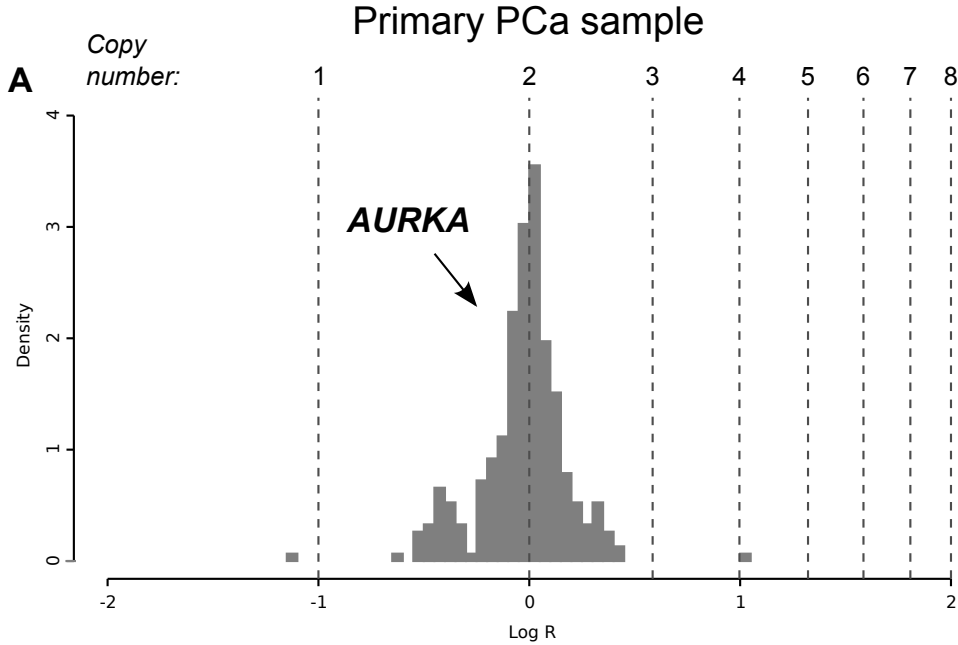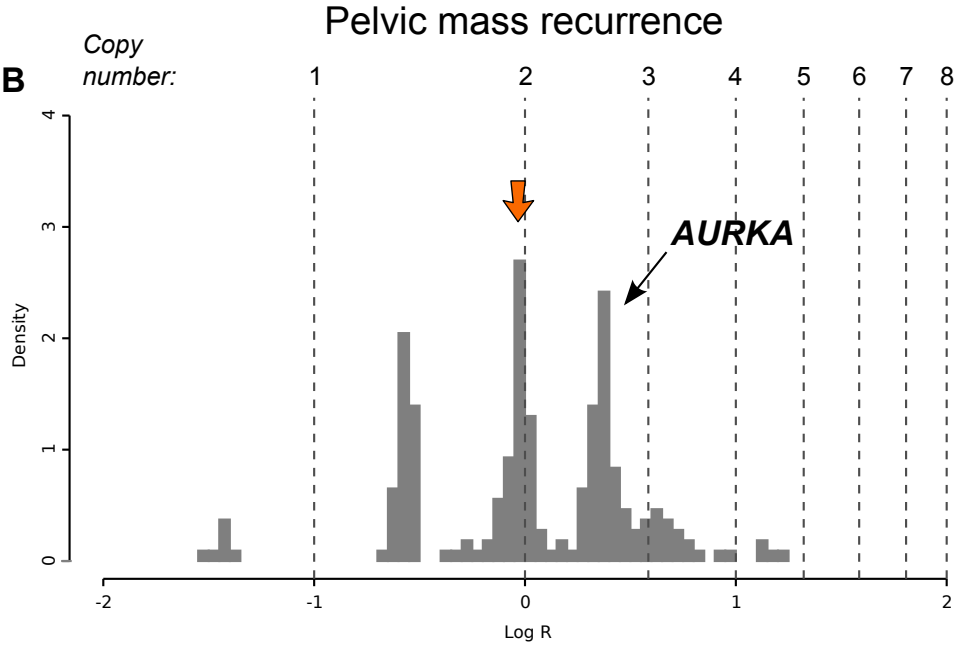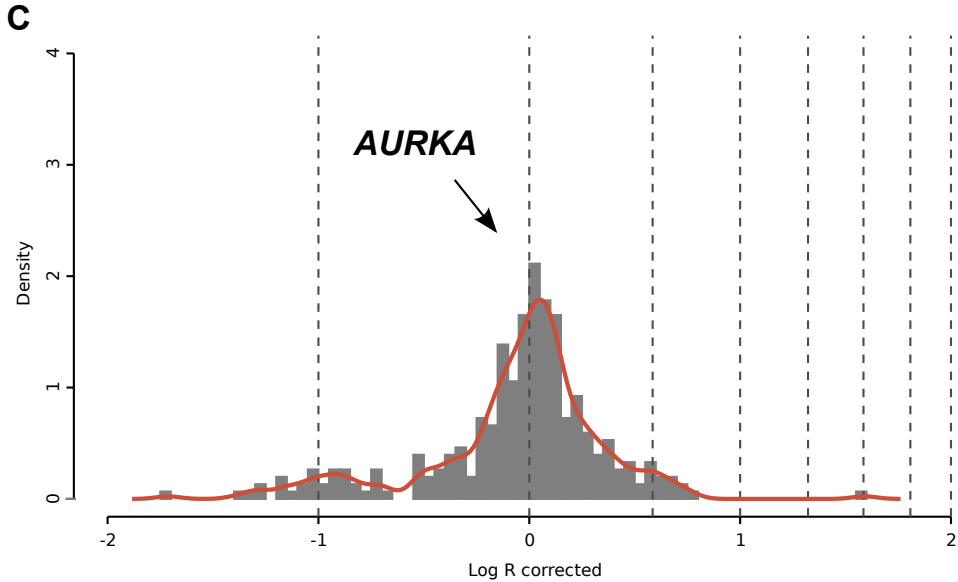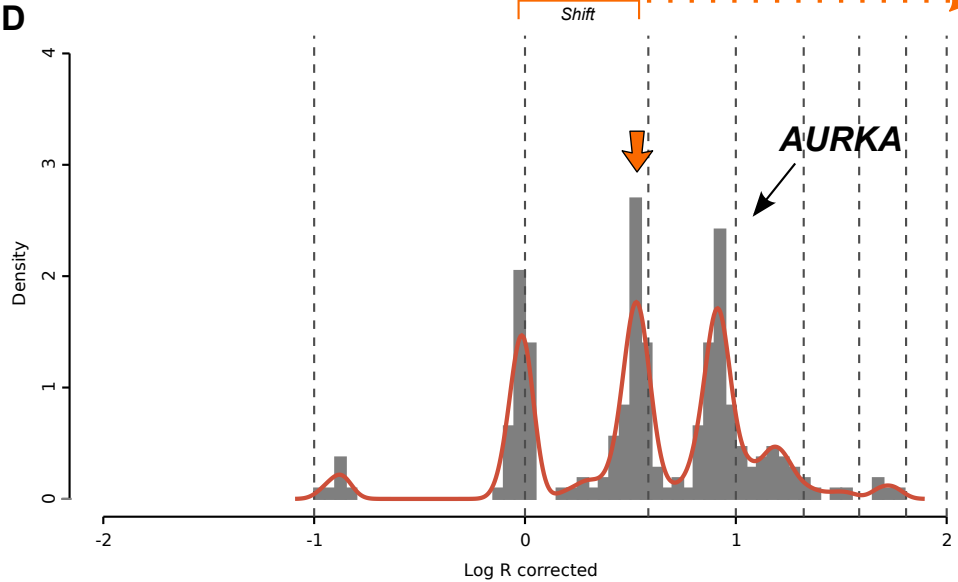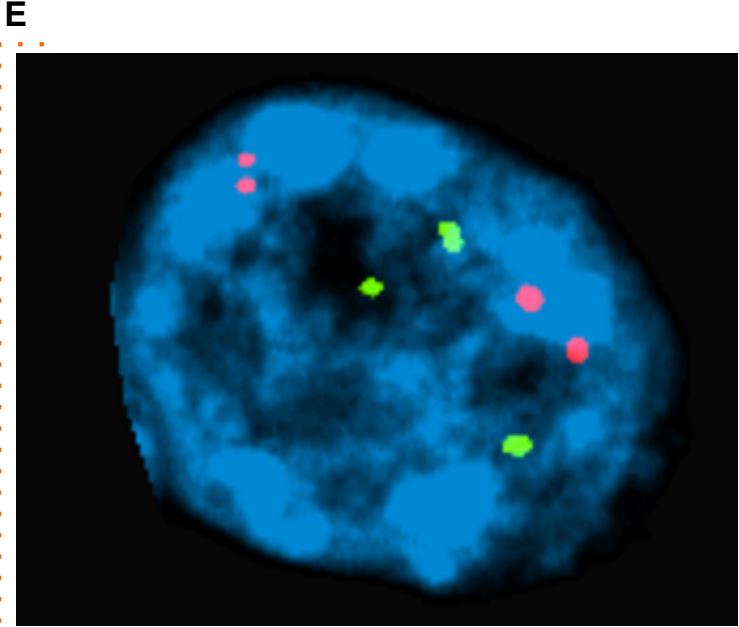

Supplement: Supplementary file 11 — Additional file 11: Figure S9.: Case of tumor progression. (A,B) The top part of the figure shows histograms of the Log R data for a primary prostate sample (A) and a pelvic mass metastasis (B) from the same patient. (C,D) Upon correction for ploidy and global admixture, CLONET identifies gene AURKA as copy number neutral in the primary sample (C) but found a gain of two copies in the late metastatic sample (D). (E) The shift in the Log R values prior and after CLONET ploidy correction in the metastatic sample indicates an aneuploidy genome, as confirmed by FISH analysis that demonstrate four yellow signals (reference probe) in tumor cells. The probes that were used for FISH assays are as follows: red test probe, 3β ERG (BAC RP11-24A11); reference probe, 10q25 (BAC RP11-431P18). (PDF 132 KB) [file 13059_2014_439_MOESM11_ESM.pdf]

# Supplementary Figure 10

**A**

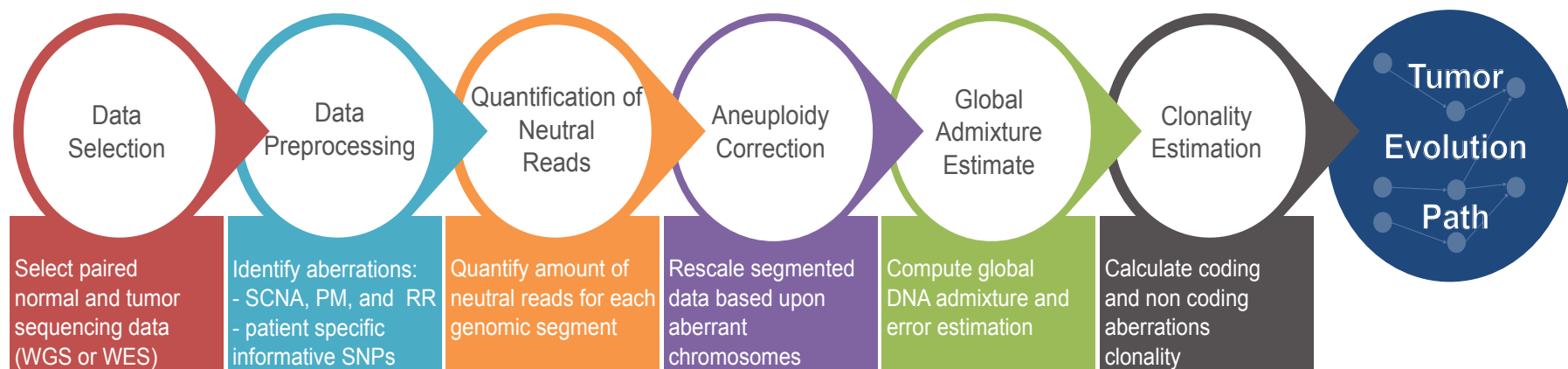

**B**

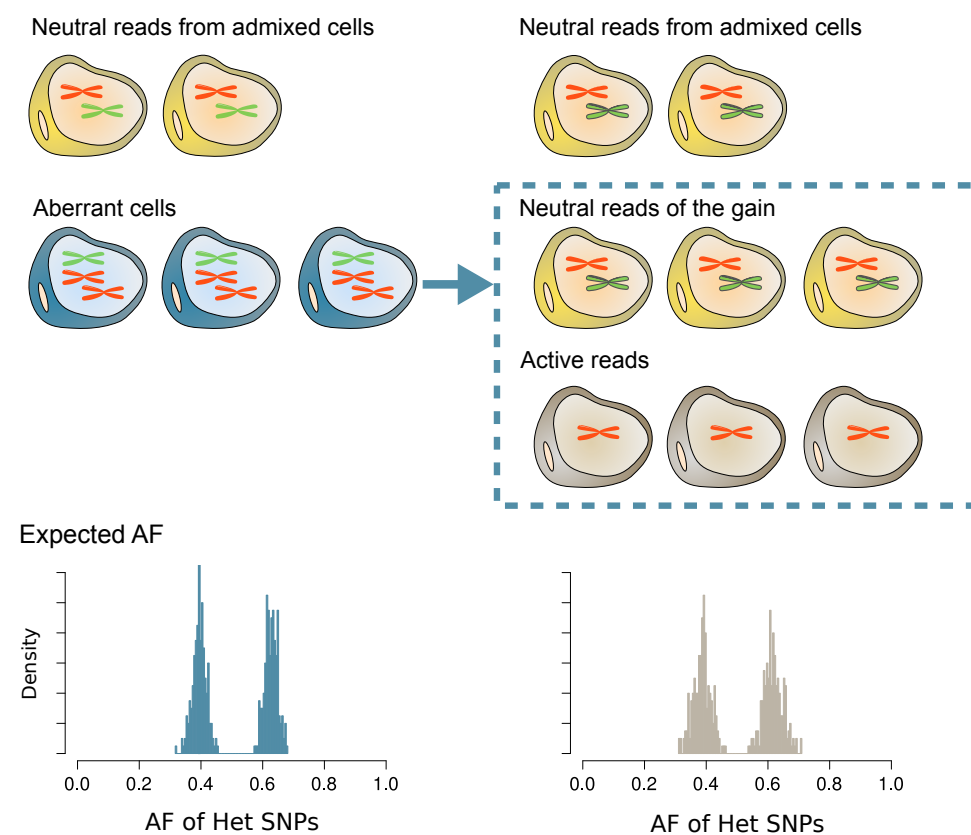

**C**

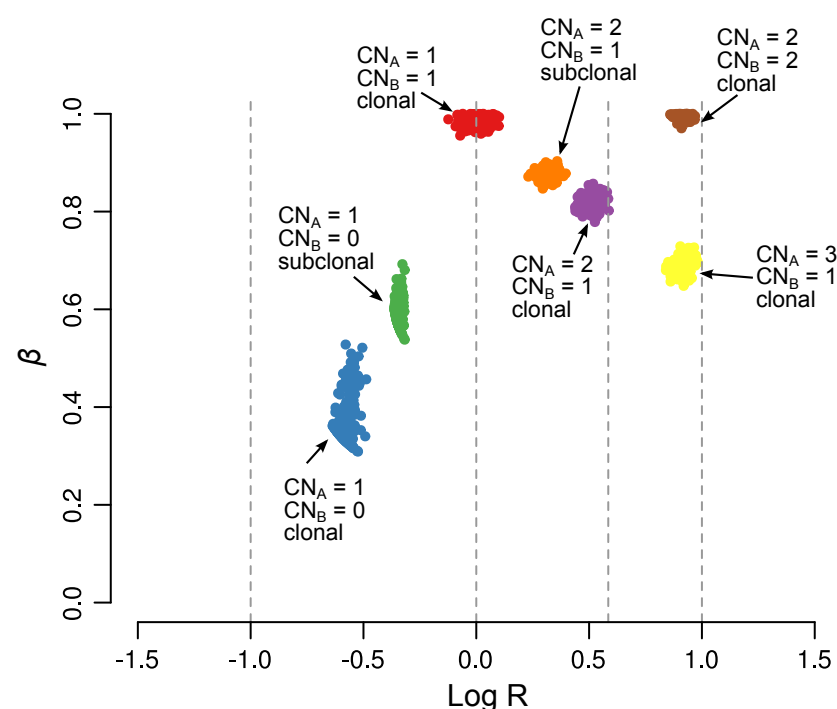

**D**

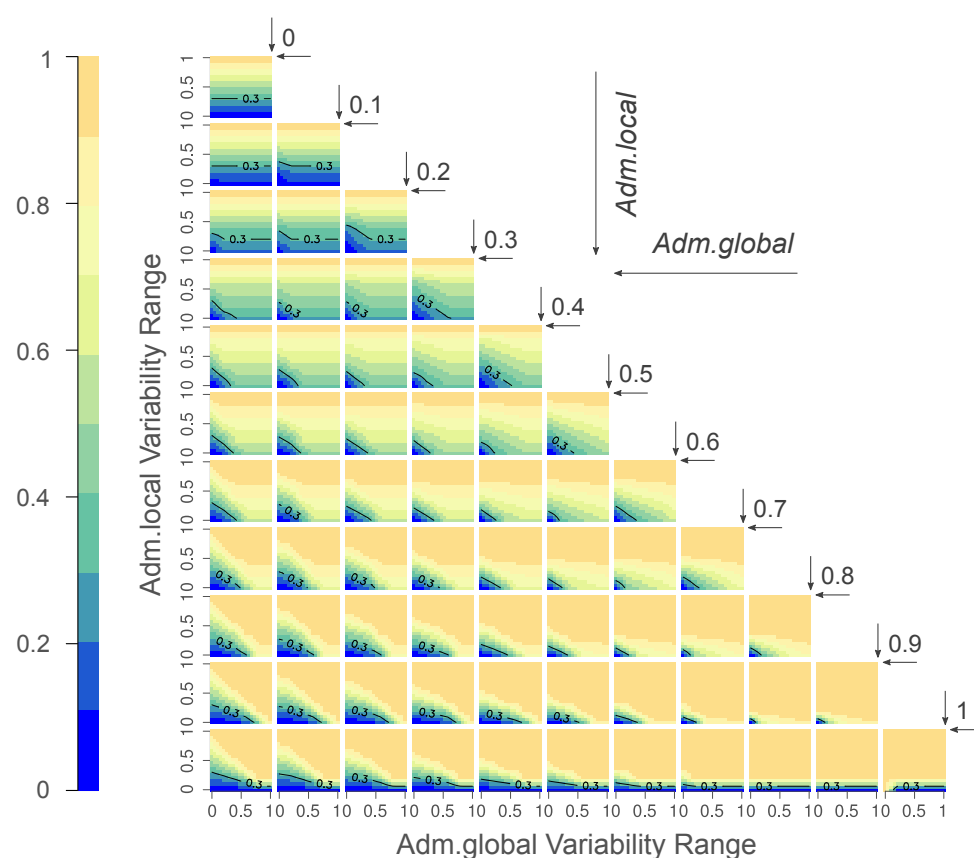

Supplement: Supplementary file 12 — Additional file 12: Figure S10.: CLONET method. (A) Schematic overview of the computing steps that lead to the definition of the tumor evolution path. (B) An example of a tumor specimen with two non-aberrant cells (yellow) and three aberrant cells (blue) with a duplicated genomic region (red). The Adm.global of this specimen is 2/5 and the percentage of aberrant reads is 4/13. Note that these values respect Equation 1. The left shows tumor cells that result from decomposing the blue aberrant cells into three normal cells and three aberrant cells with a mono-allelic deletion (brown). The percentage of neutral reads is 10/13. The value of β is rescaled to account for the gain by considering the proportion of aberrant reads is three times greater, that is, 1 - (3*(1 - β)). The bottom plot highlights that the AF of the tumor specimen and of its decomposition are the same. (C) Example of the distribution of the expected β versus Log R values in a sample with 20% of Adm.global and a mean ploidy of 2. Each point represents a genomic segment defined by its Log R value, computed by segmentation, and its β value, computed by CLONET. In particular, the blue cluster includes segments where only one allele is present in 100% of the tumor cell population (that is, they are mono-allelic clonal deletions). These segments are used to compute the Adm.global of the sample. The variability range of the Adm.global returned by CLONET considers the dispersion of the data in this cluster. (D) The plot shows how the variability ranges of local and global DNA admixture estimates propagate to the clonality values. Each box corresponds to a pair of local and global DNA admixture values and illustrates the clonality variability range as a function of their variability ranges. Local and global admixture variability ranges are computed from the β uncertainty table. (PDF 719 KB) [file 13059_2014_439_MOESM12_ESM.pdf]

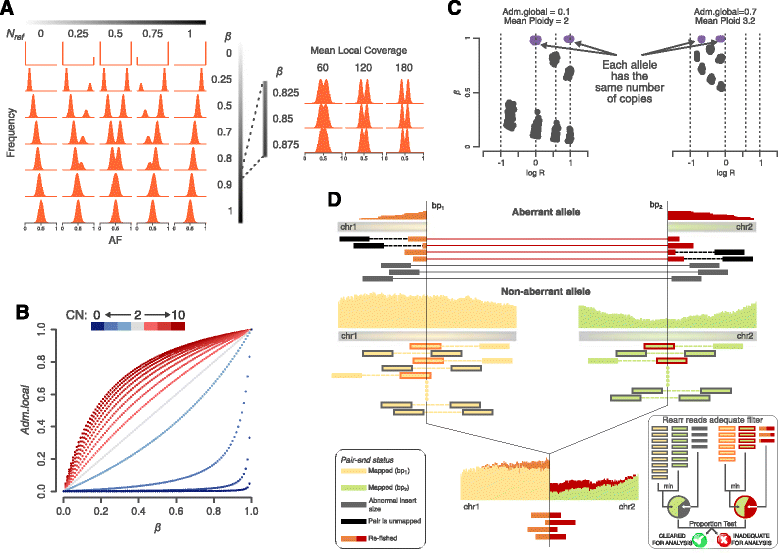

Supplement: Supplementary file 13 — Authors’ original file for figure 1 [file 13059_2014_439_MOESM13_ESM.gif]

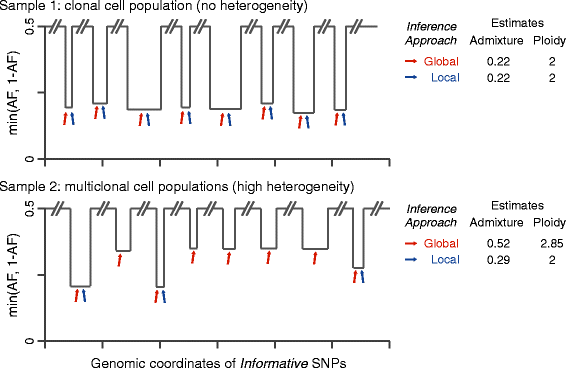

Supplement: Supplementary file 14 — Authors’ original file for figure 2 [file 13059_2014_439_MOESM14_ESM.gif]

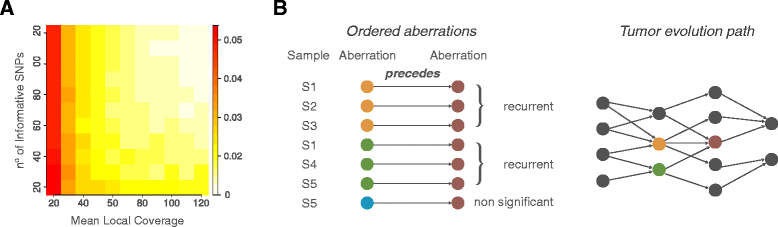

Supplement: Supplementary file 15 — Authors’ original file for figure 3 [file 13059_2014_439_MOESM15_ESM.gif]

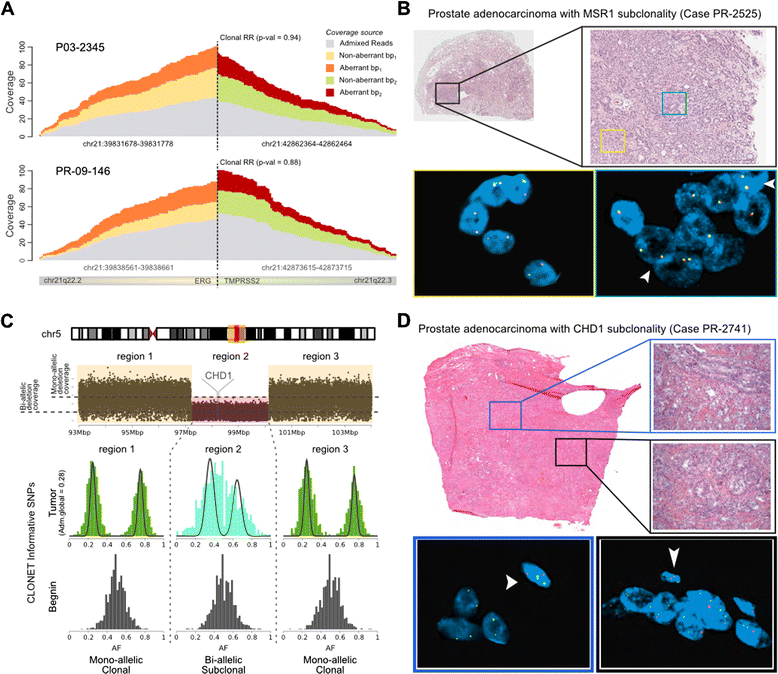

Supplement: Supplementary file 16 — Authors’ original file for figure 4 [file 13059_2014_439_MOESM16_ESM.gif]

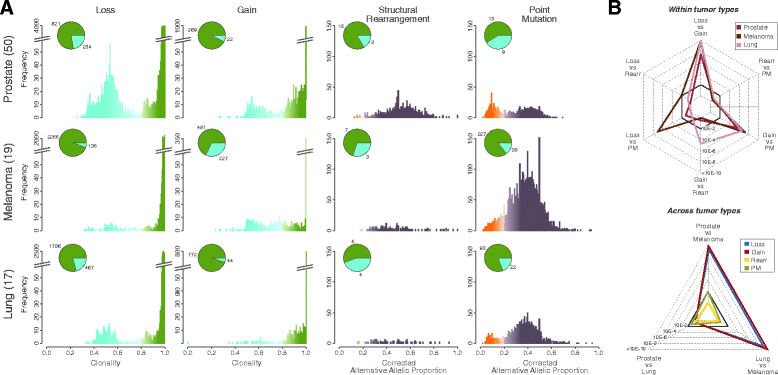

Supplement: Supplementary file 17 — Authors’ original file for figure 5 [file 13059_2014_439_MOESM17_ESM.gif]

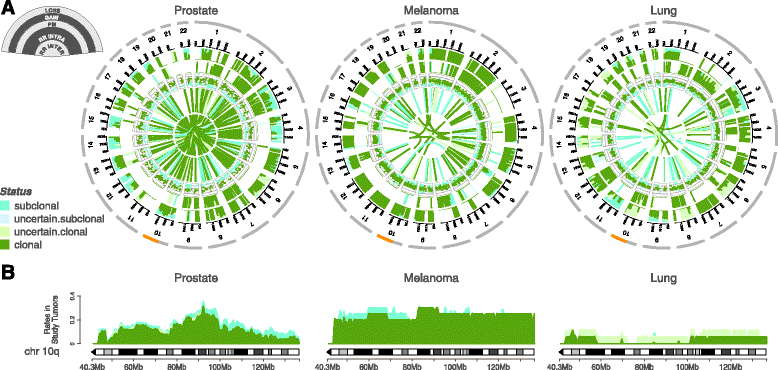

Supplement: Supplementary file 18 — Authors’ original file for figure 6 [file 13059_2014_439_MOESM18_ESM.gif]

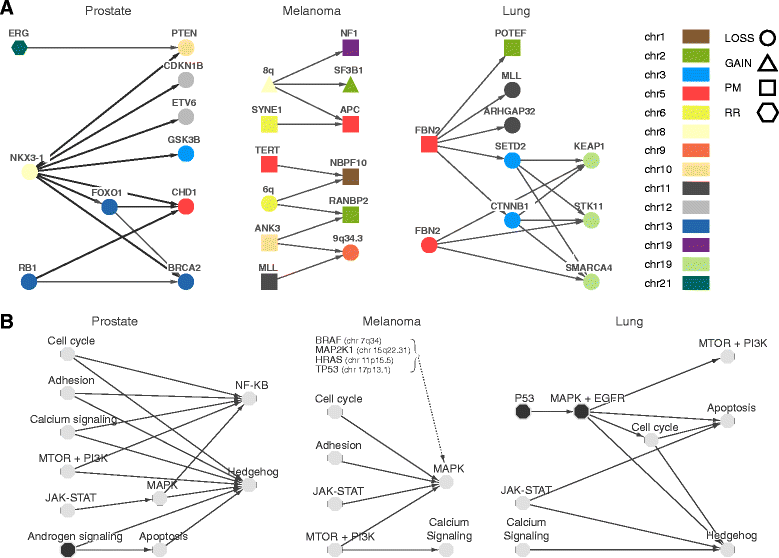

Supplement: Supplementary file 19 — Authors’ original file for figure 7 [file 13059_2014_439_MOESM19_ESM.gif]
